# Organosulfur compounds as dual-action agents: a critical review of antimicrobial and immunomodulatory potentials, and translational barriers

**DOI:** 10.3389/fphar.2026.1782223

**Published:** 2026-03-30

**Authors:** Victor Uchenna Chigozie, Lilian Nwanneka Ebenyi, Charles Okechukwu Esimone

**Affiliations:** 1 David Umahi, Federal University of Health Sciences, Ebonyi, Nigeria; 2 Pharmaceutical Microbiology and Biotechnology, Olivia University, Bujumbura, Burundi; 3 NaPBiTReG (Natural Products Bioprospecting and Translation Research Group), International Institute for Pharmaceutical Research and Innovation (IIPRI), Ebonyi, Nigeria; 4 Biotechnology, Ebonyi State University, Abakaliki, Nigeria

**Keywords:** antimicrobial resistance, dual-action therapeutics, immunomodulation, one health, organosulfur compounds, pharmacokinetics and formulation

## Abstract

The rise of antimicrobial resistance (AMR) demands therapeutic innovations that not only kill pathogens but also disarm their virulence and bolster host defenses. Organosulfur compounds (OSCs), with their unique integration of antimicrobial and immunomodulatory properties, offer a promising solution. Organosulfur compounds (OSCs) are emerging as dual-action candidates with antimicrobial and immunomodulatory potential, yet their translational readiness remains uneven across subclasses. This critical review synthesizes evidence published between 2010 and 2025 identified through targeted searches of PubMed Central, ProQuest, MDPI, ScienceDirect, and ClinicalTrials.gov, focusing on mechanistic, pharmacokinetic, and One Health–relevant dimensions. OSCs exert antimicrobial effects through thiol-reactive redox disruption, quorum-sensing inhibition, and suppression of virulence-associated transcriptional networks, with several agents demonstrating low-micromolar activity *in vitro* and measurable biofilm attenuation in preclinical models. Immunomodulatory actions—including Nrf2 activation and NF-κB suppression—are most consistently reported for isothiocyanates such as sulforaphane, which also possesses the strongest human pharmacokinetic dataset (peak plasma conjugate concentrations typically 0.one to two μM; urinary excretion >70% within 24 h). In contrast, thiosulfinates like allicin exhibit potent *in vitro* activity but are chemically unstable and seldom detectable *in vivo*, limiting systemic applicability without targeted delivery. Comparative assessment highlights significant evidence gaps: (i) absence of validated pharmacodynamic biomarkers for most OSC subclasses; (ii) limited structural optimization to mitigate off-target thiol reactivity; (iii) lack of controlled clinical trials evaluating infection-related outcomes; and (iv) scarce environmental fate data relevant to One Health frameworks. Together, these gaps underscore that OSCs should not be positioned as near-term therapeutic agents but as mechanistically rich leads requiring stabilization, standardized formulations, and biomarker-driven early-phase studies. This review provides a roadmap for advancing OSCs toward evidence-based antimicrobial and immunoregulatory interventions.

## 1 Introduction

The increasing threat of antimicrobial resistance (AMR) poses a serious challenge to global health, necessitating urgent and coordinated efforts to mitigate its impact (Chigozie VU. et al., 2025). Antimicrobial resistance (AMR) continues to evolve at a pace that outstrips current drug-development pipelines, underscoring the need for agents capable of modulating both microbial viability and host inflammatory responses (Acharya et al., 2023; Salam et al., 2023). Recent clinical and translational studies emphasize that pathogens exploit not only antibiotic resistance mechanisms but also host immune dysregulation to persist and disseminate (Lian et al., 2025; Thakur et al., 2019; Kim J. K. et al., 2025). This has shifted attention toward dual-action therapeutics—molecules that combine direct antimicrobial effects with host-targeted immunomodulation. However, many proposed dual-action agents, including phenolics and alkaloids, exhibit inconsistent potency, low bioavailability, or narrow mechanistic range, limiting their translational potential (Daley and Cordell, 2021; De Rossi et al., 2025).

Recent outcome data in multidrug-resistant Gram-negative infections emphasize that even state-of-the-art β-lactam/β-lactamase inhibitor (BL/BLI) regimens have important residual limitations. In a multicenter observational study of 183 patients with carbapenem-resistant Gram-negative bacterial infections treated with ceftazidime/avibactam-based regimens, Zhuang et al. reported a clinical response rate of 75.4%, 7-day microbiological efficacy of 43.7%, bacterial clearance of 66.0%, and 30-day all-cause mortality of 11.5% (Zhuang et al., 2023). While these data confirm that BL/BLI combinations can improve outcomes compared with legacy polymyxin-based salvage therapy, they also show that pathogen eradication is often incomplete and mortality remains substantial. Contemporary reviews likewise highlight that multidrug-resistant Gram-negative infections continue to carry high global morbidity and mortality despite the licensing of multiple new β-lactams and BL/BLI combinations, and that resistance to these agents is already emerging in clinical practice (Macesic et al., 2025; Liu et al., 2025; Russo et al., 2024; Hickson et al., 2025). Together, these observations suggest that incremental refinements in antibiotic chemistry alone are unlikely to resolve the therapeutic deficit, and motivate interest in dual-action strategies—such as organosulfur compounds—that simultaneously debulk pathogens and modulate host inflammatory and redox pathways to improve clinical outcomes and potentially slow resistance selection.

Organosulfur compounds (OSCs) represent a unique chemical class within this landscape because their sulfur-based electrophilic centers and tunable redox properties enable simultaneous interference with microbial physiology and modulation of host signaling pathways. Unlike phenolics, whose antimicrobial effects often rely on membrane disruption or nonspecific antioxidant activity, OSCs readily form reversible or irreversible adducts with cysteine-rich microbial proteins, disrupting thiol homeostasis, quorum sensing, and energy metabolism at physiologically relevant concentrations (Borlinghaus et al., 2021; Loi et al., 2015; Kim et al., 2021). Alkaloids, though biologically active, typically act through receptor binding or DNA intercalation and rarely display the broad multi-targeted reactivity characteristic of OSCs.

Beyond antimicrobial actions, OSCs such as allicin, sulforaphane, and ajoene have demonstrated potent immunomodulatory activity in both *in vitro* and *in vivo* models. Their electrophilic sulfur groups directly modify Keap1, activating the Nrf2 cytoprotective pathway, while concurrently suppressing NF-κB-mediated inflammatory cascades—an effect not consistently observed in phenolic or alkaloid scaffolds (Egbujor et al., 2022; Andrés et al., 2024; Ulasov et al., 2022). Importantly, several OSCs achieve these immunomodulatory outcomes at micromolar concentrations compatible with feasible human dosing, suggesting higher translational readiness than many other natural dual-action candidates (Baralić et al., 2024; Saito et al., 2025). Given these distinctive chemical and biological attributes, OSCs are emerging as promising candidates in dual-action antimicrobial therapy, offering a mechanistically rich platform that integrates redox-based antimicrobial effects with targeted immune modulation.

### 1.1 Novelty and contribution of this review

**FIGURE 1:**
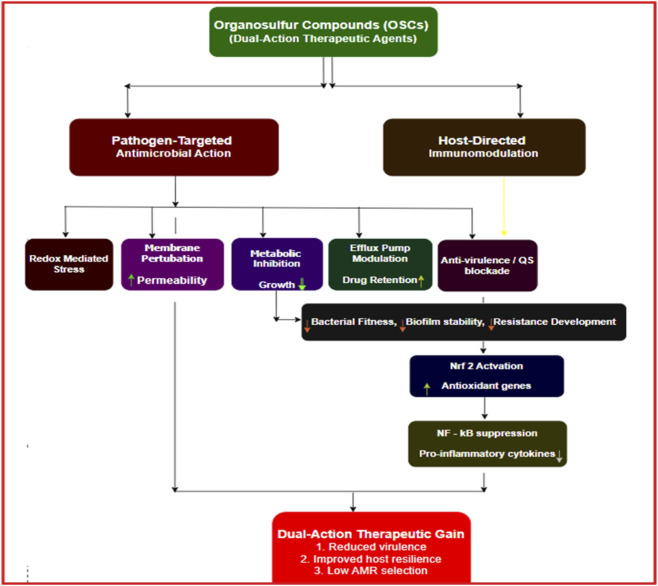
Flowchart (dual-axis: host vs. pathogen) summarizing the dual-action model OSCs, and corresponding evidence levels - OSCs exert pathogen-targeted antimicrobial effects through redox-mediated stress, membrane perturbation, metabolic inhibition, efflux pump modulation, and anti-virulence/quorum-sensing interference, supported by strong in vitro and moderate-to-strong in vivo evidence. In parallel, host-directed immunomodulation occurs via Nrf2 activation and NF-κB suppression, with robust in vitro and in vivo validation and emerging human PK/PD biomarker support, most convincingly for sulforaphane. Integration of these mechanisms reduces bacterial fitness, biofilm stability, and resistance development, culminating in dual-action therapeutic gains characterized by reduced virulence, improved host resilience, and lower antimicrobial resistance selection pressure. Evidence strength reflects the current literature across *in vitro, in vivo*, and limited human exposure–response studies.

This review critically provides a *mechanism-first, translational synthesis* of organosulfur compounds (OSCs) that is distinct from previous OSC reviews, which are typically organized by botanical source (e.g., *Allium* vs. *Brassica*) or emphasize *in vitro* antimicrobial potency alone. Rather than grouping compounds by plant origin, we classify OSCs by sulfur-chemical class and electrophilic/reactive behavior (thiosulfinates, isothiocyanates, lipophilic disulfides, synthetic analogues) and map these classes onto defined antimicrobial and immunomodulatory mechanisms. This framework is visualized in a dual-axis host–pathogen schematic (Figure 1), which explicitly links sulfur chemistry to redox-mediated antimicrobial stress, virulence suppression, and Nrf2/NF-κB immune rebalancing, providing a mechanistically coherent basis for dual-action therapy. Second, this review foregrounds *pharmacokinetic/pharmacodynamic (PK/PD) readiness and biomarker support,* rather than treating all OSCs as equally tractable. We highlight sulforaphane as an exemplar with robust human PK/PD data—including mercapturic-acid metabolites, LC–MS/MS–quantified conjugates, and Nrf2 target gene induction—and contrast this with the instability and limited systemic detectability of thiosulfinates such as allicin. We systematically link chemical stability, metabolic fate, and delivery strategies (local vs. systemic, encapsulation, controlled release) to realistic clinical scenarios, and we summarize these dimensions in a translational-readiness matrix comparing natural and synthetic OSC subclasses. Third, we situate organosulfur compounds (OSCs) within contemporary translational and safety frameworks, addressing gaps in prior reviews by explicitly linking mechanistic evidence to realistic clinical use scenarios. Rather than positioning OSCs as stand-alone antimicrobials, we frame them as adjunctive agents with plausible utility in settings where conventional antibiotics show persistent limitations—such as multidrug-resistant pneumonia, topical and device-associated biofilm infections, and gut-localized infections where high local exposure and immunomodulatory effects may be advantageous. By contextualizing OSC activity alongside current last-line antibiotic regimens (e.g., β-lactam/β-lactamase inhibitor combinations) and recent outcome data from multidrug-resistant Gram-negative infections, we underscore that incremental antibiotic optimization alone continues to leave substantial residual mortality and incomplete microbiological clearance, thereby strengthening the rationale for adjunctive, dual-action strategies. From a safety perspective, we critically evaluate OSC immunomodulation using principles established for host-directed therapies, emphasizing the need for biomarker-guided exposure, immune balance assessment, and off-target profiling. This framework treats OSCs as pharmacologically active host-modulating agents—rather than benign nutraceuticals—and outlines pragmatic early-phase monitoring considerations appropriate to their redox-reactive chemistry. Fourth, this review integrates *synthetic biology and multi-omics explicitly as enabling platforms,* not as speculative applications. We outline how microbial biosynthesis can stabilize and standardize OSC analogues, how proteomics and metabolomics can deconvolute on- and off-target S-thiolation and biotransformation, and how transcriptomics and metagenomics can monitor immunologic and One Health–relevant impacts, including resistome dynamics. These tools are presented as part of a structured translational pipeline that moves OSCs from descriptive mechanistic observations toward quantifiable, safety-aware development decisions. Finally, the review adopts a *One Health and cross-sectoral lens* that extends beyond human therapeutics. We synthesize emerging data on OSC-rich preparations in livestock and aquaculture as antibiotic-sparing feed additives, consider environmental fate and microbial ecology, and recommend targeted, monitored applications to minimize unintended ecological selection pressures.

Together, these elements—mechanism-based classification, PK/PD and biomarker emphasis, explicit clinical and immunotherapy safety benchmarking, multi-omics-enabled translational pipeline, and One Health integration—differentiate this article from prior OSC reviews and provide a roadmap from mechanistic promise to clinically and ecologically responsible dual-action application.

## 2 Methodology

### 2.1 Literature search strategy

This review was conducted as a critical narrative synthesis aimed at integrating mechanistic, pharmacological, and translational evidence on organosulfur compounds (OSCs). A narrative approach was selected because OSC research spans heterogeneous experimental domains—including microbial assays, host–pathogen interaction studies, animal infection models, pharmacokinetic/pharmacodynamic (PK/PD) investigations, limited human biomarker studies, and emerging One Health data—rendering formal meta-analytic synthesis inappropriate. Literature searches were performed across PubMed Central, ScienceDirect, ProQuest, MDPI, and ClinicalTrials.gov, covering publications between 2010 and 2025, with the final search completed on December of 2025. Searches were restricted to peer-reviewed articles published in English. Search terms were combined using Boolean operators and included keywords related to OSC chemistry (e.g., *organosulfur compounds, thiosulfinate, isothiocyanate, allicin, ajoene, sulforaphane*), antimicrobial and anti-virulence activity, redox signaling, thiol reactivity, quorum sensing, biofilm modulation, immunomodulation (e.g., *Nrf2, NF-κB*), antimicrobial resistance, PK/PD biomarkers, and One Health relevance. Reference lists of key primary studies and recent reviews were manually screened to identify additional relevant publications.

### 2.2 Eligibility criteria

Studies were eligible for inclusion if they provided experimental, mechanistic, pharmacological, or translational data relevant to the antimicrobial, anti-virulence, host-directed immunomodulatory, PK/PD, or ecological effects of OSCs. Priority was given to studies that included quantitative antimicrobial endpoints (e.g., MICs, biofilm assays), mechanistic validation (e.g., thiol modification, redox perturbation, signaling pathway engagement), *in vivo* infection or inflammation models, or human PK/PD or biomarker data. Studies were excluded if they were non-experimental commentaries, lacked sufficient methodological detail, focused solely on industrial or agricultural processing without biological relevance, or were preprints without peer review. Articles describing OSC chemistry or structure–activity relationships were included when they directly informed biological mechanism or translational feasibility.

### 2.3 Data extraction and synthesis

From each eligible study, data were extracted on OSC class and chemical features, biological system (microbial species, host cell type, animal model, or human cohort), exposure conditions, and key mechanistic endpoints (e.g., S-thiolation, membrane disruption, quorum-sensing inhibition, Nrf2/NF-κB modulation). Where available, PK/PD parameters, biomarker readouts, and safety-relevant findings were also captured. Approximately 480 records were initially identified across databases. Following title/abstract screening and full-text eligibility assessment, ∼210 studies were included in the qualitative synthesis. Given the narrative design, conclusions were not based on frequency of reporting but on convergence of evidence across independent experimental systems. Where conflicting or heterogeneous findings were observed, discrepancies were interpreted in relation to compound stability, chemical reactivity, exposure duration, biological model, and analytical methodology, rather than resolved through selective exclusion. This approach allowed mechanistic consistencies and translational constraints to be distinguished from model- or context-specific variability. Accordingly, the conclusions of this review emphasize mechanistic plausibility, biological coherence, and translational readiness, rather than claims of established clinical efficacy.

For clarity, qualitative descriptors of evidence strength (such as shown in Figures 1, 6) used in this review are defined as follows and are intended to aid interpretation across heterogeneous study designs rather than imply formal clinical grading: Strong *in vitro* evidence: reproducible effects shown in ≥2 independent studies using quantitative, standardized assays (e.g., MIC, time–kill, biofilm or quorum-sensing assays) across multiple microbial strains or species. Moderate-to-strong *in vivo* evidence: effects demonstrated in at least one well-controlled animal model, supported by quantitative microbial endpoints and/or relevant host-response measures, with greater weight given to replicated findings. Emerging human PK/PD biomarker support: controlled human studies showing measurable exposure and pathway-linked pharmacodynamic markers, without infection-related efficacy outcomes. Where evidence did not meet these criteria or was context-dependent, this is stated explicitly.

## 3 Pharmacological classes and sources of organosulfur compounds

Organosulfur compounds (OSCs) comprise a chemically diverse group of sulfur-containing molecules found across botanical, microbial, and synthetic origins. Unlike traditional classifications based on source or nomenclature, a mechanistic framework more accurately reflects their pharmacological behavior. OSC activity is largely determined by sulfur oxidation state, electrophilicity, and the capacity to form reversible or irreversible covalent interactions with thiol-containing proteins in pathogens or host cells (Pan et al., 2025; Barrett et al., 2012). This section categorizes OSCs according to dominant mechanistic classes—redox-active thiol modifiers, enzyme-targeting electrophiles, membrane-active lipophilic OSCs, and structurally stabilized synthetic derivatives—highlighting associated pharmacodynamic (PD) and pharmacokinetic (PK) attributes.

### 3.1 Redox-active OSCs (thiol–disulfide modulators)

Redox-active OSCs constitute the most chemically reactive subset of organosulfur compounds, driven by their intrinsic ability to undergo rapid thiol–disulfide exchange with cysteine residues on microbial proteins. Compounds such as allicin, diallyl disulfide (DADS), diallyl trisulfide (DATS), and ajoene exert broad-spectrum antimicrobial activity by oxidizing low–low-molecular-weight thiols (e.g., glutathione) and disrupting protein thiol networks essential for DNA synthesis, respiration, and quorum sensing (Nakamoto et al., 2020). Advanced proteomic studies show that allicin modifies >100 cysteine-containing proteins in *Staphylococcus aureus*, targeting pathways involved in translation, energy metabolism, and virulence factor expression (El-Saadony et al., 2024; Loi et al., 2019). From a pharmacokinetic standpoint, these OSCs exhibit rapid decomposition in biological fluids, contributing to short systemic half-lives. Recent metabolic profiling demonstrates that allicin and its congeners convert quickly into sulfonate and sulfoxide metabolites with diminished antimicrobial potency (Borlinghaus et al., 2021). These PK limitations underscore why clinical applications of redox-active OSCs are currently most promising in topical, gastrointestinal, or mucosal delivery systems rather than systemic formulations.

### 3.2 Enzyme-targeting electrophilic OSCs (cysteine-reactive small molecules)

**FIGURE 2:**
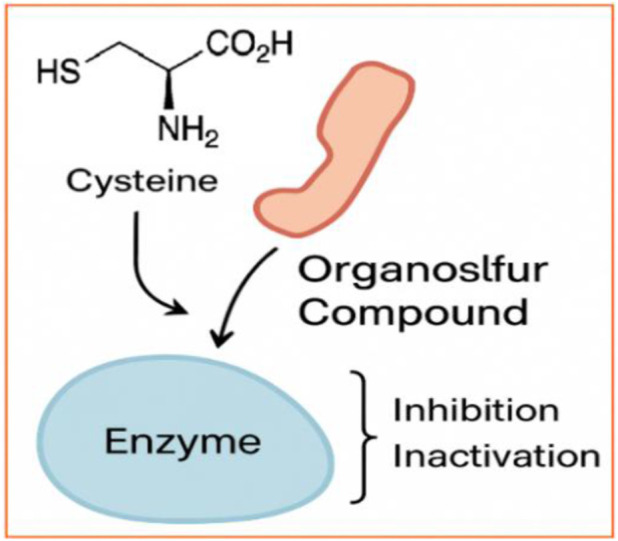
Thiol modification - Enzyme-targeting electrophilic OSCs (e.g., sulforaphane, BITC) covalently modify cysteine residues in microbial enzymes, inhibiting dehydrogenases and redox regulators (created by authors).

Electrophilic OSCs, including sulforaphane (SFN), benzyl isothiocyanate (BITC), and phenethyl isothiocyanate (PEITC), possess Michael acceptor–type functional groups capable of forming covalent adducts with nucleophilic cysteines in both microbial and host proteins (Figure 2). In pathogenic bacteria, these compounds inhibit thiol-dependent dehydrogenases, disrupt glutathione metabolism, and suppress virulence regulator systems such as SoxR and OxyR (Dagah et al., 2024). In mammalian systems, sulforaphane’s electrophilicity allows selective modification of cysteine residues in Keap1, promoting Nrf2 activation and subsequent induction of cytoprotective genes, including HO-1 and NQO1 (de Groot et al., 2022). Unlike redox-active garlic OSCs, electrophilic OSCs possess well-characterized pharmacokinetics. Clinical trials show sulforaphane achieves quantifiable plasma levels (0.5–2 µM) after oral dosing and displays a half-life of 2.2–3 h with consistent metabolite formation through glutathione conjugation (Yan and Yan, 2023). These properties provide a strong mechanistic basis for dual antimicrobial–immunomodulatory effects, making electrophilic OSCs attractive candidates for translational development.

### 3.3 Membrane-active lipophilic OSCs

Lipid-soluble OSCs, such as long-chain thioethers, thiosulfonates, and S-methyl-L-cysteine derivatives, interact primarily with microbial and mitochondrial membranes. These compounds integrate into lipid bilayers, increasing membrane fluidity, altering proton gradients, and impairing respiratory enzyme complexes (Wu et al., 2023). Recent biophysical studies using lipid vesicle models demonstrate that thioethers cause concentration-dependent increases in membrane permeability, facilitating the leakage of essential ions and metabolites (Munusamy et al., 2020).

Their enhanced lipophilicity also improves intracellular penetration, enabling multi-target actions beyond the membrane, including interference with bacterial ATP synthesis and oxidative phosphorylation. PK studies indicate longer retention in lipid-rich tissues and slower metabolic clearance compared with hydrophilic OSCs (Nikou et al., 2022). These characteristics contribute to broader antimicrobial potential but require careful toxicity evaluation due to possible mitochondrial membrane interactions.

### 3.4 Structurally stabilized synthetic OSC derivatives

Synthetic OSCs represent a growing frontier in sulfur-based drug discovery. Stabilized sulfoxides, sulfonyl derivatives, and controlled-release thiosulfinates are engineered to modulate electrophilicity, improve stability, and enhance selectivity. These compounds overcome the rapid degradation and inconsistent potency seen in natural OSCs while retaining key sulfur-dependent biological activities (Tang et al., 2025). Medicinal chemistry studies reveal that modifying the oxidation state or substituents on sulfur centers significantly alters binding affinity toward microbial thiol enzymes and redox regulators (Andres et al., 2023). Some synthetic sulfonyl analogues demonstrate potent inhibition of multidrug-resistant Gram-negative pathogens by simultaneously impairing efflux pump activity and disrupting cysteine-dependent metabolic pathways (Noor et al., 2025; Zhang et al., 2023). Improved aqueous solubility, longer half-lives, and tunable cytotoxicity profiles make synthetic OSCs promising for systemic formulations and combination therapies.

### 3.5 Natural sources vs. synthetic origins

**FIGURE 3:**
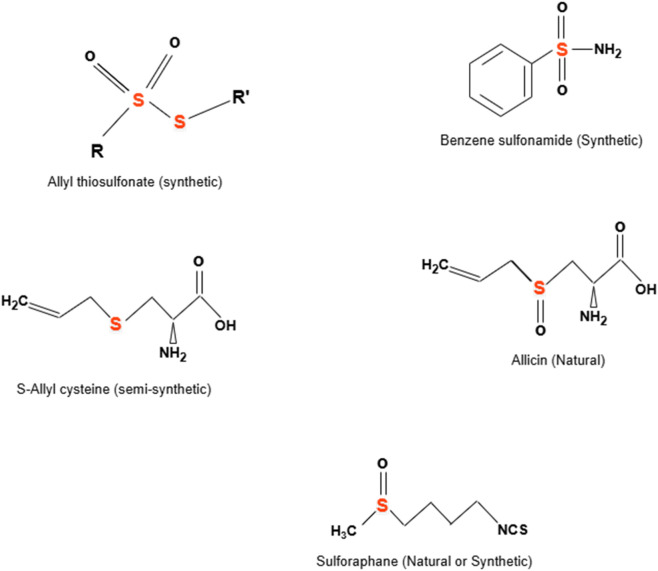
Representative chemical structures of some natural, semi-synthetic, and synthetic organosulfur compounds (OSCs), highlighting the sulfur-centered functional motifs that underlie their antimicrobial and redox-active properties.

Natural OSCs predominantly arise from *Allium* (e.g., garlic, onion) and *Brassica* (e.g., broccoli, cabbage) species through enzymatic precursors such as alliin (allicin) and glucoraphanin (sulforaphane). Their biological effects often reflect ecological defense strategies against microbial stress and herbivory (Borlinghaus et al., 2021). Synthetic OSCs expand this natural chemical space by enabling structural precision, controlled reactivity, and improved metabolic profiles. Structures of key natural and synthetic OSCs are shown below (Figure 3). Unlike source-based classifications, distinguishing OSCs by mechanism—redox-active, electrophilic, membrane-active, or stabilized synthetic—is more scientifically relevant, as pharmacological behavior depends primarily on sulfur chemistry rather than botanical origin. Table 1 categorizes OSC subclasses based on sulfur chemistry and mechanism, linking pharmacology to translational feasibility and safety considerations.

**TABLE 1:** Mechanistic OSC classification—origin, pharmacology, translational maturity, and safety considerations.

Mechanistic class	Representative OSCs	Natural vs. Synthetic	Expanded pharmacodynamic mechanisms	Key PK/PD attributes	Clinical stage	Toxicology status	Supporting literature
Redox-active thiol modulators	Allicin, DADS, DATS, Ajoene	Natural (Allium)	Thiol oxidation; disruption of glutathione homeostasis; inactivation of cysteine-rich proteins (translation/respiration); quorum-sensing inhibition	Highly reactive; rapid decomposition; short systemic half-life; poor systemic exposure	Topical/local use only; no controlled PK-guided human trials	Safety concerns linked to host thiol modification; no standardized chronic toxicity datasets; hepatotoxicity risk at high doses (preclinical evidence only)	Noor et al., 2025; Murugaiyan et al., 2022
Enzyme-targeting electrophiles	Sulforaphane (SFN), Benzyl-ITC (BITC), Phenethyl-ITC (PEITC)	Natural (Brassica), Synthetic analogues	Covalent cysteine modification; inhibition of microbial dehydrogenases; suppression of redox regulators (SoxR/OxyR); Keap1–Nrf2 immune modulation	Stable; quantifiable plasma exposure; validated mercapturic-acid PK biomarkers; reversible PD signaling	Human exposure studies ongoing (Phase 1 dietary trials, biomarker-defined dosing)	Generally well tolerated; low systemic toxicity; no long-term data; immunomodulation requires monitoring of host competence	Janczewski et al., 2022; Baralić et al., 2024
Membrane-active lipophilic OSCs	S-methyl-L-cysteine; sulfur-lipophilic thioethers	Natural & semi-synthetic	Membrane insertion and depolarization; permeability increase; oxidative respiratory chain inhibition; ATP depletion	High tissue penetration; longer tissue retention; lipophilic accumulation requiring dose control	Preclinical only — antimicrobial and antioxidant animal models	Organ-level toxicity is possible due to membrane perturbation and accumulation; dose–response not standardized	Wardecki et al., 2023; Herzog et al., 2012
Stabilized synthetic OSCs	Sulfonyl and sulfoxide derivatives; stabilized thiosulfinates	Synthetic	Tunable electrophilicity; enhanced target selectivity; efflux pump inhibition; translation-coupled anti-biofilm activity	Improved chemical stability; extended half-life; enhanced solubility; optimized PK possible	Preclinical lead identification; some showing clinical candidate potential as adjuvant efflux modulators	Limited toxicity data; requires proteomic mapping of off-target thiol interactions and *in vivo* dose-limiting toxicity studies	Bellucci et al., 2024; Beleva et al., 2025; Byrd et al., 2021

## 4 Antimicrobial mechanisms of organosulfur compounds

Because the core chemistry of organosulfur compounds (OSCs) is detailed in Section 2, the following discussion focuses on how these properties translate into pathogen-directed antimicrobial effects. Through thiol reactivity and redox modulation, OSCs disrupt microbial membranes, inactivate enzymes, impair metabolism, and interfere with communication networks. Compounds such as allicin, thiosulfinates, and thiosulfonates exhibit broad-spectrum antibacterial, antiviral, and antifungal activity—including against resistant Gram-positive and Gram-negative pathogens—via mechanisms that include oxidative stress induction, membrane perturbation, efflux modulation, and anti-virulence effects (Guillamón et al., 2021; Rouf et al., 2020). Integrating these mechanistic pathways with quantitative and molecular data provides a framework for understanding OSCs as dual-action antimicrobial agents, as explored in the sections that follow.

### 4.1 Redox-mediated antimicrobial stress

Redox-active organosulfur compounds (OSCs), including allicin, diallyl disulfide (DADS), diallyl trisulfide (DATS), and ajoene, exert antimicrobial activity by inducing oxidative stress through thiol–disulfide exchange, causing widespread S-thiolation of cysteine residues in microbial proteins. This disrupts redox homeostasis and impairs essential processes such as metabolism, DNA replication, and protein synthesis (Percio et al., 2024; Boutin et al., 2024). Proteomic analyses show that allicin modifies over 100 proteins in *Staphylococcus aureus*, consistent with MICs of 16–64 μg/mL for *S. aureus* and 64–256 μg/mL for *Pseudomonas aeruginosa*, while DADS and DATS display comparable activity across Gram-positive and Gram-negative bacteria (Tang et al., 2021).

### 4.2 Enzyme inhibition and metabolic disruption

Electrophilic organosulfur compounds (OSCs) such as sulforaphane, benzyl isothiocyanate (BITC), and phenethyl isothiocyanate (PEITC) inhibit microbial growth by covalently modifying cysteine residues in redox and detoxification enzymes, thereby impairing metabolic regulation and stress tolerance (Dong et al., 2024; Li W. et al., 2020). These same electrophilic interactions activate host Nrf2 signaling *via* Keap1 modification, linking antimicrobial and immunomodulatory effects (Unoki et al., 2020). Consistent with this mechanism, sulforaphane inhibits *E. coli* and *Salmonella enterica* at 64–128 μg/mL, while BITC and PEITC show greater potency against Gram-positive bacteria (8–64 μg/mL) (Romero-Durán et al., 2024).

### 4.3 Membrane perturbation

**FIGURE 4:**
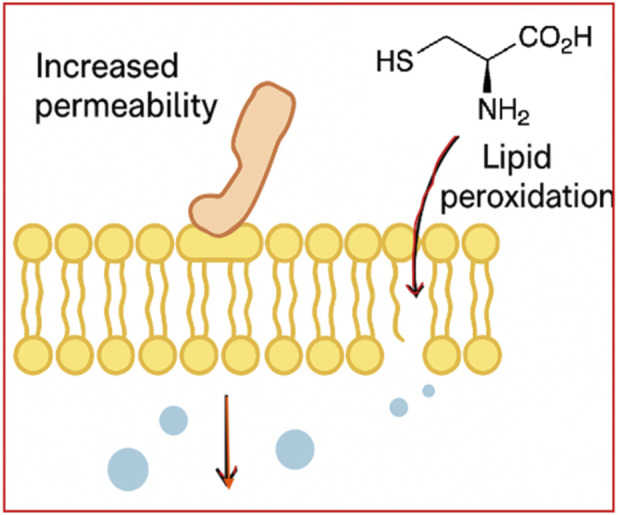
Membrane Disruption by organosulfur compounds - Membrane-active OSCs (lipophilic thioethers) insert into lipid bilayers, increasing permeability, collapsing proton gradients, and impairing ATP synthesis (created by authors).

Lipophilic organosulfur compounds (OSCs), including S-methyl-L-cysteine derivatives and long-chain thioethers, insert into bacterial membranes, increasing fluidity and disrupting bilayer integrity, proton gradients, and ATP synthesis (Shin et al., 2024; Hadjicharalambous et al., 2022). Membrane permeability and depolarization occur in a concentration-dependent manner, particularly in Gram-negative bacteria such as *E. coli* and *Pseudomonas aeruginosa* (32–128 μg/mL) (Gbala et al., 2022). Allicin and diallyl disulfide exhibit similar membrane-disruptive activity across multidrug-resistant Gram-positive and Gram-negative pathogens, underscoring membrane perturbation as a key antimicrobial mechanism of lipophilic OSCs (Table 2). Figure 4 provides a visual summary of membrane-targeting OSCs, highlighting the multi-target effects of lipophilic OSCs on bacterial membranes and intracellular energy metabolism.

**TABLE 2:** Representative antimicrobial organosulfur compounds (OSCs) with experimentally validated mechanisms, model-specific activity, and evidence for antibiotic synergy.

OSC (class)	Primary antimicrobial mechanism(s)	Representative pathogen/model	Evidence type	Synergy with antibiotics	Selected references
Allicin (thiosulfinate)	Electrophilic S-thiolation of cysteine-rich enzymes; disruption of cytoplasmic and membrane redox homeostasis; increased membrane permeability; antibiofilm activity *via* inhibition of cyclic-di-GMP–regulated thiol enzymes	MDR respiratory pathogens and Gram-negatives, including *Pseudomonas aeruginosa*, *Klebsiella pneumoniae*, and *Staphylococcus aureus*; planktonic and biofilm models	*In vitro* MIC and time-kill assays; biofilm and vapor-phase killing models	Synergizes with β-lactams against *S. aureus* and *P. aeruginosa* *in vitro*; potentiates domiphen against biofilm formation, reducing biomass and viable counts versus monotherapy	Borlinghaus et al., 2014; Li et al., 2024; Choo et al., 2020
Ajoene (disulfide/ajoene derivatives)	Quorum-sensing interference (LasR/RhlR) and anti-virulence effects (↓pyocyanin, elastase, biofilm matrix); ROS-linked stress and membrane perturbation at higher concentrations	*P. aeruginosa* PAO1 and clinical isolates; *Mycobacterium* spp. Biofilm and planktonic models	*In vitro* quorum-sensing, biofilm, and ROS assays; preclinical biofilm models	Demonstrates enhanced antibiofilm and antimycobacterial activity when combined with conventional agents *via* efflux modulation and QS inhibition; formal checkerboard synergy with specific antibiotics remains emerging	Jakobsen et al., 2012; Sarangi et al., 2024; Xie et al., 2022
Diallyl disulfide (DADS, polysulfide OSCs)	Redox stress *via* ROS generation; S-thiolation and inhibition of metabolic enzymes; modulation of efflux (e.g., norA downregulation); disruption of membrane potential	*Candida albicans* intestinal infection models; *S. aureus* and enteric bacteria	*In vitro* growth and membrane-potential assays; *in vivo* murine gut infection and protection models	Mechanistic rationale for synergy through efflux pump modulation and redox stress is strong, but systematic antibiotic-combination studies are still limited; currently best viewed as a candidate efflux-modulating adjuvant	Song et al., 2021; Hu et al., 2022; Igwe et al., 2025
S-allyl cysteine (SAC, water-soluble OSC)	Modest direct antimicrobial effects; interference with toxin production (e.g., inhibition of *S. aureus* α-hemolysin); reduction of fimbrial adhesion and colonization *via* effects on surface structures and host–pathogen interactions	*S. aureus* (α-hemolysin), uropathogenic *E. coli* (fimbrial adhesion), and gut pathogens	*In vitro* toxin and adhesion assays; cell-based colonization models	No robust, standardized synergy datasets yet; anti-virulence and adhesion-blocking actions suggest potential as a non-bactericidal adjunct that could reduce required antibiotic exposure	Kosuge, 2020; Yang et al., 2022; Sarshar et al., 2020
Sulforaphane (SFN, isothiocyanate OSC)	Electrophilic modification of microbial and host thiols; inhibition of type III secretion systems; impairment of intracellular survival and colonization; interference with metabolic and redox enzymes	*Salmonella enterica* intracellular infection models; broad Gram-positive and Gram-negative panels	*In vitro* MIC and intracellular survival assays; murine infection models	Combination data with classic antibiotics are limited but emerging; redox and secretion-system targeting supports its development as an adjunctive intracellular-active agent	Danish Rizvi et al., 2025; Pötschke et al., 2025
Synthetic sulfonyl/sulfonamide OSC analogues	Inhibition of multidrug efflux systems (e.g., AcrAB–TolC); disruption of cysteine-dependent metabolic pathways; membrane and permeability effects depending on scaffold	MDR *E. coli* and other Gram-negatives with overexpressed AcrAB–TolC; plant and human pathogens in efflux-focused models	*In vitro* MIC and accumulation assays; transporter-knockout and overexpression strains	Potentiate activity of multiple antibiotic classes by increasing intracellular drug accumulation and lowering MICs; strong candidate scaffold for rational antibiotic-adjuvant design	Byrd et al., 2021; Pun et al., 2023; Noor et al., 2025

### 4.4 Efflux pump modulation

**FIGURE 5:**
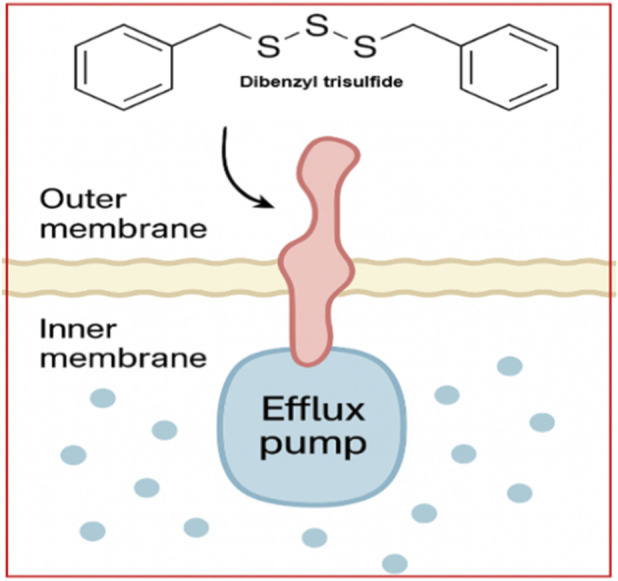
Efflux pump modulation by OSCs - Efflux pump inhibition further reduces microbial survival and pathogenicity by downregulating transporter systems (e.g., NorA, AcrAB-TolC) (created by authors).

Bacterial efflux pumps are key drivers of multidrug resistance by lowering intracellular antibiotic concentrations. Several organosulfur compounds (OSCs) modulate these systems, thereby enhancing antimicrobial efficacy. Synthetic sulfonyl OSC analogues inhibit the AcrAB–TolC efflux pump in *E. coli*, increasing intracellular antibiotic accumulation, while diallyl disulfide suppresses *norA* expression in *Staphylococcus aureus*, reducing fluoroquinolone efflux (Byrd et al., 2021; Igwe et al., 2025). These mechanistic findings support the use of OSCs as antibiotic adjuvants, as illustrated in Figure 5, highlighting efflux inhibition as a contributor to their multi-target antimicrobial activity.

### 4.5 Anti-virulence and quorum-sensing interference

Because biofilm disruption, quorum-sensing interference, and anti-virulence activities arise from overlapping biochemical interactions, these processes are consolidated here to avoid mechanistic redundancy.

**FIGURE 6:**
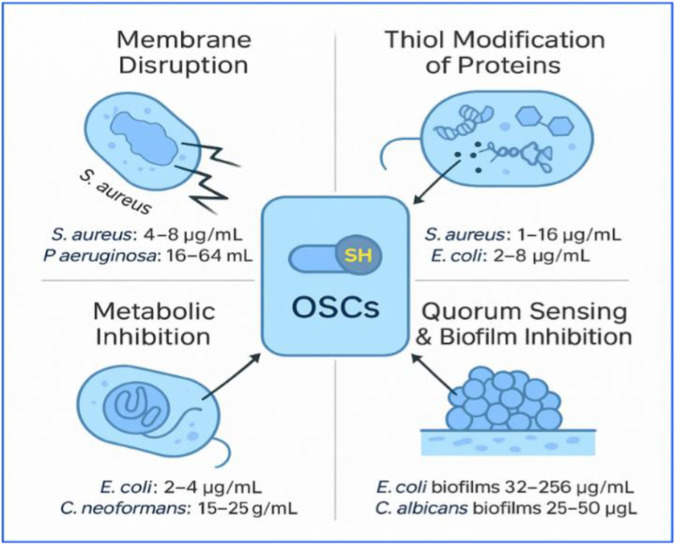
Core antimicrobial mechanisms of organosulfur compounds (OSCs) with representative MIC ranges (created by authors).

Organosulfur compounds (OSCs) not only exhibit direct antimicrobial effects but also attenuate bacterial pathogenicity through anti-virulence mechanisms (by selectively targeting virulence factors essential for host colonization, immune evasion, and disease progression) without necessarily killing microbes, which reduces selective pressure for resistance. Mechanistically, (Figure 6), OSCs can neutralize toxins *via* S-thiolation of secreted bacterial proteins or by modifying host cell receptors, thereby mitigating toxin-mediated cytotoxicity (Federic et al., 2025). Ajoene inhibits *the quorum-sensing regulators LasR and RhlR* in *Pseudomonas aeruginosa*, reducing pyocyanin production, elastase activity, and biofilm formation (Jakobsen et al., 2012; Xie et al., 2022). Structural precision of signal molecules, which usually ensures specific activation of their respective receptors, LasR and RhlR, facilitating the production of metabolites (pyocyanin, rhamnolipids, elastase), which are essential for infection establishment, is impaired by OSCs (Chigozie et al., 2025a). Ajoene represents a leading anti-virulence OSC, reducing virulence factor expression and biofilm formation (Table 2), aside from other methods such as precision phototherapies (Ding et al., 2025c; Ding et al., 2025b), and pH-Responsive AIE Photodynamic therapies (Ding et al., 2025a). Furthermore, S-allyl cysteine has been shown to inhibit *Staphylococcus aureus* α-hemolysin activity and reduce *Escherichia coli* fimbrial adhesion to epithelial cells, impairing critical steps in host attachment and invasion (Kosuge, 2020; Yang et al., 2022). Allicin suppresses biofilm matrix synthesis by inhibiting cyclic-di-GMP–regulated thiol-dependent enzymes (Reiter et al., 2017). Sulforaphane interferes with the type III secretion system in *Salmonella*, preventing maturation of effector proteins and host-cell invasion (Danish Rizvi et al., 2025). Figure 6 further depict these anti-virulence mechanisms, among others. By disarming rather than killing bacteria, these anti-virulence strategies exert reduced selective pressure for resistance development—a principle increasingly embraced in antimicrobial stewardship.

OSCs act through four validated antimicrobial mechanisms: (1) membrane disruption, producing permeability defects (e.g., *S. aureus* 4–8 μg/mL; *P. aeruginosa* 16–64 μg/mL); (2) thiol modification, where electrophilic OSCs oxidize or S-thiolate cysteine-rich enzymes in translation, respiration, and redox pathways (*S. aureus* 1–16 μg/mL; *E. coli* 2–8 μg/mL); (3) metabolic inhibition, impairing central metabolism and energy generation (*E. coli* 2–4 μg/mL; C. neoformans 15–25 μg/mL); and (4) quorum-sensing and biofilm suppression, reducing cross-cell communication and biofilm biomass (*E. coli* 32–256 μg/mL; C. albicans 25–50 μg/mL). Together, these multi-target actions underpin the broad-spectrum antimicrobial activity of OSCs.

All antimicrobial mechanisms shown are supported by strong *in vitro* evidence across Gram-positive, Gram-negative, and fungal pathogens, with quantitative MIC and biofilm data. *In vivo* support exists for redox-mediated killing, biofilm reduction, and virulence attenuation in animal models, though effects are compound and context-dependent. Human PK/PD evidence remains limited and indirect, currently strongest for sulforaphane through exposure–biomarker correlations rather than direct antimicrobial outcomes.

### 4.6 Integrative mechanistic perspective

The antimicrobial efficacy of organosulfur compounds (OSCs) reflects **multi-modal synergy**, in which redox stress, enzyme inhibition, membrane perturbation, efflux modulation, and virulence attenuation act in concert to enhance bacterial killing and limit resistance development. Synergistic combinations—such as allicin with sulforaphane—can increase bactericidal activity by more than 50% compared with individual agents, illustrating how integrated mechanisms outperform single-target effects (Choo et al., 2020; Li et al., 2024; Pötschke et al., 2025). This holistic framework positions OSCs as translationally attractive dual-action agents, with Table 2 summarizing key chemical classes, mechanisms, pathogen targets, and antibiotic synergy potential.

Importantly, these antimicrobial effects intersect with host immunity rather than acting in isolation. By modulating immune signaling, redox balance, and inflammatory pathways, OSCs can enhance immune-mediated pathogen clearance while limiting collateral tissue damage. Garlic-derived OSCs activate neutrophil calcium flux and ROS production, while allicin and isothiocyanates regulate NF-κB signaling and cytokine output, promoting balanced immune responses (Schepetkin et al., 2019; Ruhee et al., 2020; Barman et al., 2025; Jikah et al., 2024). This host–pathogen crosstalk provides the conceptual bridge to the dual-action immunomodulatory roles discussed in the following section.

## 5 Immunomodulatory actions of organosulfur compounds

The immunomodulatory effects of organosulfur compounds (OSCs) arise from targeted interactions with redox-sensitive signaling pathways rather than nonspecific immune activation. Distinct from their antimicrobial actions on microbial thiols, OSCs reprogram host inflammatory signaling and immune–redox balance, influencing both innate and adaptive immunity *in vivo* (Schepetkin et al., 2019). Garlic-derived OSCs exemplify this activity, regulating neutrophil function and oxidative responses while suppressing excessive inflammation through inhibition of NF-κB signaling and pro-inflammatory mediators, including nitric oxide, IL-6, and TNF-α (Ruhee et al., 2020). Across animal infection models, these effects translate into enhanced lymphocyte responses, improved antibody production, and balanced Th1/Th2 polarization, with concurrent modulation of NF-κB- and MAPK-dependent pathways (Cascajosa-Lira et al., 2022; Firmino et al., 2021). Dietary supplementation studies further demonstrate improved immune competence and reduced inflammatory pathology in models of infection and chronic inflammation, although human data remain limited and largely prophylactic in scope (Rouf et al., 2020; Marefati et al., 2021; Salehi et al., 2019). Collectively, OSCs act as immune-balancing agents by coordinating redox signaling, cytokine output, and immune cell function, rather than through isolated antimicrobial effects. As summarized in Table 3, suppression of NF-κB-driven inflammation, reinforcement of endogenous antioxidant defenses *via* Nrf2, and regulation of innate immune cytokine profiles underpin the dual-action therapeutic potential of OSCs**.**

**TABLE 3:** Evidence-based immunomodulatory mechanisms of key organosulfur compounds.

OSC	Molecular target(s)	Model system	Immune pathway modulated	Observed immune outcome	References
Sulforaphane (SFN)	Keap1 Cys151/273/288	Human macrophages; LPS-mice	Nrf2–Keap1 activation	↑HO-1, NQO1; ↓TNF-α, IL-6; reduced oxidative stress	Andrés et al., 2025; Mayer et al., 2024
Benzyl/Phenethyl isothiocyanate (BITC/PEITC)	Keap1 cysteine residues; MAPKs	Airway epithelium; THP-1 cells	Nrf2 activation; MAPK inhibition	↓IL-1β, MCP-1; improved epithelial barrier integrity	Park et al., 2022; Ko et al., 2025
Allicin	IKKβ; NF-κB p65; redox-sensitive proteins	THP-1 macrophages; peritonitis mice	NF-κB inhibition; cytokine suppression	↓IL-6, IL-8, TNF-α; M2-like polarization	Kim et al., 2024; Zhang L. et al., 2025
Ajoene	IκBα phosphorylation; redox signaling	Macrophages; airway epithelial cells	NF-κB and AP-1 suppression	↓IL-8, COX-2; reduced inflammatory chemokines	Cho et al., 2019; Shang et al., 2019
Diallyl disulfide (DADS)	IKK complex; redox-modified enzymes	Murine macrophages	NF-κB inhibition	↓TNF-α, ↓IL-1β	Song et al., 2021; Zhang et al., 2020; Ouyang et al., 2025
Sulfonyl and synthetic OSC analogues	MAPKs; redox-sensitive kinases	*In vitro* macrophage inflammatory models	MAPK–AP-1 pathway suppression	↓COX-2, ↓PGE2	Kim S. Y. et al., 2025; Kim et al., 2019

### 5.1 Redox-dependent modulation *via* the Nrf2–Keap1 axis

Electrophilic organosulfur compounds (OSCs), particularly isothiocyanates such as sulforaphane, consistently exert immunoregulatory effects through activation of the Keap1–Nrf2 antioxidant pathway. By modifying redox-sensitive cysteine residues on Keap1, these compounds stabilize Nrf2, induce cytoprotective gene expression (e.g., HO-1, NQO1), and suppress oxidative stress–driven inflammation. Across human macrophage models and rodent LPS-challenge systems, sulforaphane-mediated Nrf2 activation reduces intracellular reactive oxygen species, attenuates NF-κB signaling, and lowers pro-inflammatory cytokine release, including TNF-α and IL-6 (Yan and Yan, 2023; Dewanjee et al., 2024). Animal studies further demonstrate reduced oxidative tissue injury and systemic inflammation, although inter-individual variability in human bioavailability necessitates controlled pharmacokinetic strategies for clinical translation (ElKhalifa et al., 2023). Excessive or prolonged Nrf2 activation (immunomodulation) has been associated with impaired host defense, altered immune surveillance, and adverse outcomes in certain chronic inflammatory and oncologic contexts. In addition, inter-individual variability in OSC bioavailability and redox sensitivity may amplify off-target effects with repeated or high-dose exposure. Accordingly, clinical translation should emphasize short-course or localized use, biomarker-guided dosing, and careful patient selection, ensuring that immunomodulation tempers pathological inflammation without compromising antimicrobial immunity or long-term immune homeostasis (Pouremamali et al., 2022).

### 5.2 Suppression of pro-inflammatory signaling (NF-κB and MAPKs)

Several organosulfur compounds (OSCs), including allicin, ajoene, and diallyl disulfide (DADS), attenuate canonical pro-inflammatory signaling by targeting redox-sensitive components of upstream kinase pathways. Mechanistically, S-thiolation of cysteine residues on IKKβ and related signaling proteins suppresses IκBα phosphorylation and limits NF-κB (p65) nuclear translocation, resulting in consistent reductions in IL-1β, IL-6, and TNF-α across cellular and animal models. OSCs also modulate MAPK pathways (p38, ERK), thereby dampening AP-1–dependent transcription of COX-2 and inflammatory chemokines (Song et al., 2021). These effects are reproducible *in vitro*, where macrophages exposed to pharmacologically relevant concentrations of allicin or DADS show rapid suppression of NF-κB activation and downstream cytokine release, and *in vivo*, where OSC treatment reduces neutrophilic infiltration, inflammatory signaling, and tissue pathology in rodent models of peritonitis and airway inflammation (Song et al., 2021; Hu et al., 2022). Collectively, NF-κB and MAPK modulation by OSCs supports their use as adjunctive agents in settings characterized by excessive inflammation, provided dosing strategies preserve essential host microbicidal functions**.**

### 5.3 Modulation of cytokine and chemokine profiles

Across immune models, organosulfur compounds (OSCs) consistently suppress pro-inflammatory cytokines (IL-6, IL-1β, TNF-α) while preserving or enhancing anti-inflammatory mediators such as IL-10, particularly in epithelial–immune co-culture systems and animal models of inflammatory challenge (Babiuchi et al., 2020). Sulforaphane shows especially reproducible effects, inhibiting MCP-1 and IL-6 production in airway epithelial cells and macrophages exposed to particulate pollutants, bacterial ligands, or viral proteins, with concomitant preservation of epithelial barrier integrity—effects linked to antioxidant and anti-inflammatory signaling pathways (Patel et al., 2020; Habtemariam, 2024). Related OSCs such as allicin and ajoene similarly attenuate chemokine release (e.g., IL-8, RANTES) in airway epithelial models and reduce neutrophil recruitment and tissue injury in rodent lung inflammation models (Arellano Buendía et al., 2023).

### 5.4 Functional reprogramming of immune cells (macrophages, neutrophils, epithelial cells)

Beyond pathway-level effects, organosulfur compounds (OSCs) modulate immune cell phenotype and function in a context-dependent manner. In macrophages, allicin and diallyl disulfide (DADS) promote a pro-resolution, M2-like phenotype characterized by increased IL-10 and arginase-1 and reduced iNOS expression in both stimulated primary cells and tissue macrophages *in vivo* (Fitzsimons et al., 2023). Preclinical studies further indicate that appropriate OSC dosing can preserve or enhance neutrophil phagocytic activity while regulating oxidative burst, thereby limiting collateral tissue damage without abolishing antimicrobial function, although effects vary by compound and model (Loh and Vermeren, 2022). In parallel, isothiocyanates and related OSCs reinforce epithelial barrier integrity under inflammatory stress by reducing cytokine signaling and strengthening antioxidant defenses, leading to decreased paracellular permeability in intestinal and airway models (Lai et al., 2023; Kim et al., 2023). Collectively, these functional shifts support a role for OSCs in promoting inflammation resolution while preserving essential innate defenses; however, their immunological effects are concentration- and time-dependent, and translation is constrained by limited *in vivo* human immunophenotyping data.

### 5.5 Integrated mechanistic summary and translational considerations

Taken together, these mechanisms explain how organosulfur compounds (OSCs) can attenuate pathological inflammation while preserving essential host defenses when appropriately dosed: activation of the Keap1–Nrf2 pathway limits oxidative stress and indirectly suppresses NF-κB signaling, direct S-thiolation of upstream kinases dampens pro-inflammatory transcription, and membrane- and stress-responsive effects stabilize epithelial barriers and shape immune cell phenotypes (Dewanjee et al., 2024). For translation, established pharmacodynamic endpoints—including Nrf2 target induction (HO-1, NQO1), NF-κB p65 nuclear localization, cytokine ratios (IL-6/TNF-α/IL-10), phagocyte oxidative burst, and epithelial permeability—provide measurable markers of on-target activity in early human studies (Yan and Yan, 2023). However, because OSC reactivity can lead to off-target thiol modification, clinical development requires careful concentration–response optimization and, for less stable compounds such as allicin analogues, formulation or localized delivery strategies; existing human sulforaphane studies demonstrate that such biomarker-guided dosing is feasible at tolerable exposure levels (ElKhalifa et al., 2023).

### 5.6 Benchmarking OSC immunomodulation against immunotherapy safety frameworks

Established safety frameworks for biologic immunotherapies provide a useful benchmark for evaluating organosulfur compounds (OSCs) as host-directed immunomodulators. These frameworks emphasize early safety monitoring, predictive biomarkers, immune-balance assessment, and benefit–risk stratification—principles that are equally applicable to OSC development (Zhang S. et al., 2025). Although OSCs are small molecules and thus less likely to elicit classic immunogenicity, their electrophilic sulfur chemistry introduces distinct risks related to off-target thiol modification. Redox-proteomic studies demonstrate that OSCs such as allicin and ajoene can induce widespread S-thiolation of mammalian proteins, including cytoskeletal, metabolic, and signaling components, with measurable effects on immune signaling and cellular integrity (Gruhlke et al., 2019; Mösbauer et al., 2021). These findings underscore the need for systematic off-target profiling during preclinical development.

From a translational perspective, OSC-mediated immunomodulation—principally *via* Nrf2 activation and NF-κB suppression—offers therapeutic benefit in hyperinflammatory states but may compromise host defense if dosing, duration, or clinical context are not carefully controlled (Fagian et al., 2020; Egbujor et al., 2022). Accordingly, early-phase studies should incorporate dose–response characterization, infection-competence assessments, and longitudinal safety monitoring, including immune phenotyping and organ toxicity markers, particularly if chronic or prophylactic use is envisaged (Onisei et al., 2023; Guo et al., 2025). Patient stratification based on immune status, comorbidities, microbiome composition, and concomitant redox-active exposures will be essential to optimize safety and efficacy. Aligning OSC development with established immunotherapy safety principles, therefore, provides a rigorous, evidence-based pathway to responsibly advance these compounds as host-directed adjuncts rather than empiric immunomodulators.

## 6 Dual-action synergy

Organosulfur compounds (OSCs) share a unified chemical basis for dual-action therapy, with sulfur electrophilicity driving both pathogen-directed effects and host-directed immunomodulation through redox and inflammatory pathways. While preclinical evidence strongly supports this mechanistic convergence, human validation remains limited—largely confined to biomarker studies of sulforaphane—highlighting the need for cautious, stepwise clinical translation.

### 6.1 Evidence for mechanistic synergy (summary of recent data)

Studies from 2010 to 2025 provide consistent evidence for dual-action effects of organosulfur compounds, showing concurrent suppression of pathogen burden and host inflammation. Examples include ajoene reducing *Pseudomonas* quorum sensing and lung inflammation, allicin–isothiocyanate combinations enhancing bacterial killing while lowering cytokine release, and sulforaphane linking systemic exposure to Nrf2 activation with reduced intracellular pathogen survival (Jakobsen et al., 2012; Alibrahem et al., 2025; Gong et al., 2024). Together, these findings indicate that selected OSCs can achieve improved composite outcomes compared with antimicrobial or anti-inflammatory monotherapy.

### 6.2 Translational priorities and lead-compound selection

To translate mechanistic promise into clinically viable candidates, we propose a streamlined, evidence-based prioritization cascade. First, PK/PD tractability should be decisive, favoring OSCs with reproducible human or large-animal exposure biomarkers or stable synthetic derivatives; sulforaphane exemplifies this class due to validated LC–MS/MS assays and Nrf2 pharmacodynamic markers enabling exposure–response modeling in early trials. Second, candidates should demonstrate dual-action efficacy across orthogonal systems, combining direct antimicrobial activity (standardized planktonic and biofilm assays) with measurable host-directed effects in immune assays and at least one relevant infection model that quantifies both pathogen burden and tissue pathology. Third, off-target and safety liabilities must be addressed early through redox-proteomics, cytotoxicity testing in primary human cells, and short-term toxicology to exclude broadly reactive candidates. Finally, formulation feasibility should guide selection, prioritizing OSCs amenable to stabilization or localized delivery where systemic use of labile compounds is impractical**.**

### 6.3 PK/PD and biomarker strategy for dual-action studies

A dual-action PK/PD plan should quantify both exposure and effect across microbial and host axes within feasible clinical windows. *Exposure* is best assessed by LC-MS/MS measurement of the parent compound and key metabolites (e.g., SFN–NAC), with urinary mercapturic acids reflecting cumulative dosing. *Host pharmacodynamics* should include Nrf2 target induction, inflammatory cytokine ratios (IL-6/TNF-α/IL-10), and glutathione redox status, while *antimicrobial activity* is evaluated using standardized MIC/time-kill, biofilm assays, and tissue bacterial burden *in vivo*. Sampling should capture early peak exposure (≈0.5–8 h) and later downstream responses (24–48 h).

### 6.4 Formulation and delivery approaches that enable dual-action use

Because chemical instability is a key translational barrier, formulation strategy largely determines feasibility. Labile thiosulfinates such as allicin are best suited to *local delivery* (topical, inhaled, or GI-targeted) to achieve local PK/PD effects while limiting systemic off-target thiol reactivity. *Stabilized synthetic scaffolds or prodrugs* with tunable electrophilicity can retain antimicrobial activity with improved safety, while *encapsulation approaches* (e.g., lipid nanoparticles or microencapsulation) may enhance stability of compounds such as sulforaphane or protect allicin *in situ*, provided release profiles and immunogenicity are carefully controlled.

### 6.5 Preclinical study design recommendations

Before asserting dual-action potential in humans, a minimal preclinical package should demonstrate convergent microbial and host effects. This includes *in vitro* antimicrobial activity (MICs, time-kill, biofilm reduction, antibiotic synergy), *ex vivo* human immune modulation and pathogen-clearance assays, and *in vivo* validation in at least one infection model comparing OSC alone, antibiotic alone, and combination therapy, with parallel assessment of pathogen burden, host inflammation, and PK/PD–tissue exposure relationships.

### 6.6 One Health and agricultural relevance

Organosulfur compounds (OSCs) are being explored as antibiotic-sparing feed additives in livestock and aquaculture, highlighting real-world dual-action potential. However, such use requires stringent resistome surveillance and environmental fate assessment to prevent unintended selection pressures or ecological toxicity, necessitating longitudinal monitoring when deployed at scale.

### 6.7 Research gaps and prioritized next steps

Key gaps include limited validated pharmacodynamic biomarkers beyond sulforaphane, lack of standardized and stabilized delivery formulations, incomplete mapping of host proteome S-thiolation to define therapeutic windows, inconsistent combination-testing pipelines, and insufficient field data linking agricultural use to resistome outcomes. Priority actions are to advance early human PD studies for SFN-class compounds, accelerate medicinal chemistry toward stabilized OSC leads with parallel off-target profiling, and integrate One Health surveillance into all animal-use programs.

## 7 Pharmacokinetics/pharmacodynamics, and formulation considerations

Translational development of OSCs hinges on a realistic appraisal of ADME (absorption, distribution, metabolism, excretion) and on formulation strategies that reconcile chemical reactivity with safety and exposure control.

### 7.1 Chemical stability and metabolic fate

A realistic translational strategy for organosulfur compounds (OSCs) begins with a clear-eyed view of chemical stability and metabolic fate. Two distinct pharmacochemical behaviors require separate development paths: *(i). Labile thiosulfinates (e.g., allicin and related Allium-derived OSCs): -* Allicin (diallyl thiosulfinate) and closely related thiosulfinates are highly electrophilic and undergo rapid chemical transformation in aqueous and biological milieus. They react readily with low-molecular-weight thiols (glutathione, cysteine) and protein thiols, and they decompose to a mixture of polysulfides, ajoenes, and volatile sulfides. As a result, the parent allicin molecule is rarely detectable in plasma after oral ingestion of garlic; instead, downstream metabolites (e.g., allyl methyl sulfide, diallyl disulfide derivatives) and conjugates are measured. These properties create two practical consequences: (1) systemic exposure to intact allicin is limited unless the compound is chemically stabilized or delivered locally; and (2) pharmacodynamic effects may derive from short-lived parent species acting locally (e.g., in the gut or at mucosal surfaces) or from active metabolites. These phenomena are well summarized in recent reviews and primary studies of garlic bioactives (El-Saadony et al., 2024; Thakur et al., 2024). *(ii). Electrophilic isothiocyanates (e.g., sulforaphane):* Isothiocyanates such as sulforaphane (SFN) exhibit greater tractability for systemic pharmacology. After ingestion (often as glucoraphanin converted to SFN by myrosinase activity in plants or gut microbiota), SFN undergoes rapid conjugation with glutathione and sequential metabolism *via* the mercapturic-acid pathway to produce SFN–GSH, SFN–CysGly, SFN–Cys, and SFN-N-acetylcysteine (SFN–NAC), which are quantifiable in plasma and urine. This well-defined metabolic cascade provides reproducible biomarkers of exposure and target engagement (e.g., Nrf2 target gene induction). Nonetheless, SFN displays substantial inter-individual variability in systemic exposure because of differences in dietary preparation (presence/activity of plant myrosinase) and microbiome-mediated conversion, which must be characterized for clinical translation (Grady et al., 2023; Olayanju et al., 2024).

*Practical implications*: *(I)* For allicin-like OSCs, systemic therapeutic strategies should prioritize local delivery (topical, inhaled, GI-targeted) or chemical stabilization (prodrug/formulation) because of rapid parent instability. For systemic indications, consider focusing on more stable OSC derivatives or synthetic analogues (El-Saadony et al., 2024). *(II)*. For isothiocyanates (sulforaphane class), validated PK/PD biomarkers (mercapturic acid metabolites, HO-1/NQO1 induction) enable early-phase human studies to link exposure with pharmacology; however, PK studies must account for variability introduced by food matrix and microbiome (Grady et al., 2023; Olayanju et al., 2024). Encapsulation and controlled-release platforms have been proposed to address these limitations.

Given allicin’s well-documented chemical and biological instability in aqueous and lipid environments (e.g., short half-life in oil, rapid degradation) (Zhou et al., 2025). This review emphasizes formulation approaches (e.g., encapsulation, liposomal delivery) and local-delivery routes (e.g., topical, pulmonary) as practical translational strategies—rather than relying purely on *in vitro* potency.

### 7.2 Isothiocyanate pharmacokinetics—model data and PK/PD linkages

Because sulforaphane (SFN) is the best-characterized OSC in humans, it serves as a practical example for how to conduct PK/PD-informed translation: *(I). Absorption and metabolism -* Orally administered SFN (or glucoraphanin when converted to SFN) is absorbed and rapidly conjugated *via* the glutathione pathway; urinary excretion of SFN–NAC is a reliable exposure biomarker. High-sensitivity LC–MS/MS methods can quantify plasma SFN and its conjugates at low nanomolar–micromolar concentrations, enabling correlation of exposure with target engagement (Nrf2-driven gene expression) (Baralić et al., 2024; Grady et al., 2023). *(II). Representative PK parameters (typical ranges reported in controlled studies) -* Reported plasma concentration ranges and half-life estimates vary with formulation and dose, but human studies commonly observe detectable SFN/metabolites in plasma within 0.5–2 h post-dose, with major urinary excretion of mercapturic acid metabolites over the subsequent 24–48 h. Reported elimination half-life estimates for SFN conjugates are typically on the order of ∼1–4 h, depending on dose and assay sensitivity; however, exact values depend on preparation (isolated SFN vs. glucoraphanin + myrosinase) and host factors (Giron et al., 2024). These data support feasible SAD/MAD designs in Phase I-type studies with PD sampling windows within the first 8–24 h after dosing (Grady et al., 2023; Olayanju et al., 2024). (*III). PK/PD linkage -* Controlled human supplementation trials have demonstrated dose-dependent induction of Nrf2 target genes (HO-1, NQO1) in peripheral blood mononuclear cells and reduced selected inflammatory biomarkers—providing mechanistic PD endpoints that can be used to establish pharmacologically active exposure levels for future infection–adjunct trials. Because PD effects are measurable at achievable oral exposures, SFN-type compounds are suitable candidates for initial human proof-of-mechanism work (Yan and Yan, 2023; Baralić et al., 2024). Because sulforaphane is uniquely characterized by robust human PK/PD data (mercapturic-acid metabolites) and validated Nrf2 activation biomarkers, we highlight it as a priority lead for first-in-human, dual-action proof-of-mechanism studies, aligning with our translational roadmap.

### 7.3 Formulation and targeted delivery strategies

Given the divergent chemical behaviors of OSC subclasses, formulation strategies must be tailored: *(I)* **.** *Stabilization of labile OSCs (Allium thiosulfinates) -* Approaches that have been explored to preserve allicin activity include micro-encapsulation, liposomal encapsulation, nano-emulsions, and inclusion complexes. Encapsulation can limit premature thiol reactions and control release kinetics; several preclinical studies and formulation reviews report improved stability and sustained release profiles for allicin-loaded liposomes and polymeric nanoparticles. Nevertheless, formulation must be validated for *(i)* retention of biological activity after encapsulation, *(ii)* predictable release kinetics at the intended site, and *(iii)* avoidance of off-target thiol modification that could cause toxicity (El-Saadony et al., 2024; Zhang et al., 2024). *(II) Nano- and lipid-based carriers for isothiocyanates and lipophilic OSCs -* Lipid nanoparticles, solid lipid nanoparticles, and nano-emulsions improve solubility, protect sensitive moieties, and can be surface-functionalized for tissue targeting (e.g., lung inhalation, wound dressings). Recent reviews of nano-formulations for plant-derived actives demonstrate that such platforms increase bioavailability and enable local high-concentration delivery while reducing systemic exposure—attributes particularly useful when local antimicrobial or immunomodulatory action is desired. When adopting nanocarriers, developers must assess carrier-driven immunogenicity, stability, and scalable manufacturing approaches (Zhang et al., 2024; Albakr et al., 2024). *(III) Infection-site-responsive prodrugs and triggered release -* A promising translational tactic is prodrug design or triggerable carriers that respond to infection microenvironment cues—e.g., elevated ROS, acidic pH, or pathogen proteases—to release active OSCs selectively at infected sites. Although conceptually attractive, most infection-responsive OSC prodrugs are at the preclinical stage; demonstration of selective activation, local PD effect, and safety in animal infection models is required before human translation (Zhang et al., 2024; Albakr et al., 2024).

Regulatory and manufacturing considerations for formulations - Formulation strategies must be accompanied by validated analytical methods to quantify parent compound and metabolites (plasma and tissue), stability-indicating assays, and release profiling under physiological conditions. For botanical-source OSCs, standardization of precursor content (e.g., glucoraphanin for SFN, alliin/allinase activity for allicin precursors) is necessary to ensure reproducible exposure in human studies (El-Saadony et al., 2024; Grady et al., 2023).

### 7.4 Drug–drug interaction considerations and phase 1 monitoring strategy

Sulforaphane and garlic-derived thiosulfinates (organosulfur compounds, OSCs) can modulate xenobiotic metabolism by upregulating phase II detoxifying enzymes, especially glutathione-S-transferases (GSTs) and UDP-glucuronosyltransferases (UGTs), and by influencing glutathione-dependent conjugation pathways. *Induction of Phase II Enzymes*: Both sulforaphane and garlic OSCs (e.g., diallyl disulfide, diallyl trisulfide) increase the expression and activity of GSTs and UGTs in various cell types and animal models, primarily through activation of the Nrf2/ARE pathway and AP-1 transcription factors. This upregulation enhances the conjugation and elimination of xenobiotics and drugs, potentially altering their pharmacokinetics and effects (Stoian et al., 2025). *Glutathione Conjugation:* OSCs can form glutathione conjugates, directly participate in redox reactions, and modulate cellular glutathione pools, further influencing detoxification and drug metabolism (Bhuiyan et al., 2015; Zhang et al., 2013). *Selectivity and Structure-Activity:* The number of sulfur atoms and chemical structure of OSCs affect their potency in inducing phase II enzymes. For example, sulforaphane and diallyl trisulfide are more potent inducers than diallyl sulfide (Lii et al., 2010). This interaction may impact the metabolism and efficacy of conventional therapeutics (Tang et al., 2025). Although significant clinical drug–drug interactions (DDIs) have not yet been conclusively documented for most OSCs, preclinical and human biomarker studies indicate the potential for altered clearance of co-administered agents undergoing thiol-based redox cycling or Nrf2-influenced hepatic metabolism (Egbujor et al., 2022; Tang et al., 2025). Accordingly, early-phase clinical studies should incorporate DDI-focused pharmacokinetic monitoring, including co-administration of probe substrates for CYP3A4 and GST activity, serial quantification of glutathione redox status, and targeted LC–MS/MS assessment of OSC–drug metabolite conjugates. In addition, routine hematology, liver function tests, and inflammatory cytokine profiling will support real-time detection of host response modulation and unanticipated adverse reactions. This pragmatic approach enables risk-controlled clinical advancement while generating the mechanistic data necessary to guide appropriate use alongside standard antimicrobial or immunomodulatory therapies.

To contextualize the heterogeneous development status of different OSC subclasses, Table 4 summarizes their comparative translational readiness across preclinical efficacy, safety evidence, formulation maturity, and clinical evaluation.

**TABLE 4:** Comparative translational readiness of representative organosulfur compounds (OSCs).

OSC/Subclass	Preclinical efficacy (antimicrobial/Immunomodulatory)	Safety/Toxicology data	Formulation/Stability/Delivery maturity	Clinical trial status/Human data	References
Allicin (Natural (garlic) thiosulfinate)	Potent *in vitro* activity against bacteria and fungi (e.g., *Cryptococcus neoformans* MICs in the low µg/mL range) with *in vivo* efficacy in murine cryptococcosis; mechanism includes membrane damage and redox stress	Limited systematic mammalian safety data; strong thiol-reactivity implies risk of off-target protein modification; no robust chronic toxicity studies	Chemically unstable in aqueous systems; rapidly decomposes to polysulfides; no widely adopted, standardized pharmaceutical formulation—mostly crude or aged garlic preparations with variable allicin content	No registered antimicrobial or immunotherapy trials using defined allicin as a drug; human exposure primarily *via* diet or nutraceuticals with poorly standardized dosing	Li X. et al., 2022; Nakamoto et al., 2020; JIkah et al., 2024
Diallyl disulfide (DADS) and related garlic sulfides	Demonstrates antibacterial, antifungal, anti-inflammatory, and antioxidant activity *in vitro* and in animal models; some antimicrobial effects, but less potent than allicin on a molar basis	Relatively more toxicology and mechanistic data than many OSCs (anticancer and organ-protection models), but still lacking dedicated infection-focused safety packages; chronic toxicity and immunotoxicity are not comprehensively defined	More chemically stable than allicin but still not developed as a formal drug product; generally delivered as part of garlic oil or experimental preparations, not as a defined clinical formulation	No OSC-specific antimicrobial clinical trials; human relevance inferred from garlic-rich diet and cancer/organ-protection experimental contexts rather than infection-focused trials	Song et al., 2021; Mitra et al., 2022
Ajoene (garlic-derived disulfide)	Validated anti-virulence and quorum-sensing inhibitor: inhibits QS-regulated genes and biofilm formation in *Pseudomonas aeruginosa* and other pathogens; reduces virulence in animal infection models	Limited mammalian safety data; mostly short-term toxicity and cell-culture tolerability; no formal immunotoxicity or chronic safety evaluation, though doses used in preclinical models have not shown overt toxicity	Chemically more stable than allicin, synthetic ajoene analogues have been generated to improve potency and drug-like properties, but no standardized clinical formulation exists	No registered human clinical trials as an antimicrobial or anti-virulence drug; translational development remains preclinical, despite strong mechanistic proof-of-concept	Jakobsen et al., 2012; Nakamoto et al., 2020
Sulforaphane (SFN; isothiocyanate)	Moderate direct antimicrobial effects in some models; the strongest evidence lies in host-directed immunomodulation *via* Nrf2 activation and NF-κB modulation, reducing oxidative and inflammatory damage in diverse preclinical disease models	Comparatively well-characterized safety profile; multiple human studies show tolerability and Nrf2 biomarker activation (e.g., phase II enzyme induction, anti-inflammatory effects) at dietary/supplemental doses	Oral formulations (broccoli sprout extracts, stabilized SFN or glucoraphanin + myrosinase) are available and have been standardized for clinical research; metabolic fate (mercapturic acid pathway) and biomarker readouts are well described	Multiple small human trials and intervention studies in non-infectious indications (e.g., oxidative stress, metabolic and inflammatory conditions, HIV-associated inflammation), but no dedicated antimicrobial or infection-adjunct trials to date	Saito et al., 2025; Alves et al., 2025; Fahey et al., 2015; Giron et al., 2024
Other dietary isothiocyanates (e.g., PEITC, BITC)	*In vitro* antibacterial and anti-biofilm activity has been reported for several isothiocyanates; some host-directed antioxidant and anti-inflammatory effects have been observed in preclinical models, but less extensively characterized than SFN.	Safety is generally extrapolated from food intake and chemopreventive studies; specific immunotoxicity and infection-focused safety datasets are sparse	Available mainly as dietary components or experimental extracts, a few encapsulation and delivery approaches have been explored but not standardized as antimicrobial products	Limited or no infection-directed clinical trial data; some chemoprevention or metabolic-disease trials exist, but OSC-specific antimicrobial applications remain speculative	Qadri et al. (2022)
Synthetic sulfur-containing camphor derivatives	Newly synthesized camphor–sulfur derivatives show *in vitro* antibacterial and antibiofilm activity against Gram-positive and Gram-negative pathogens, often with MICs in the low-to-mid µg/mL range and biofilm inhibition at sub-MIC concentrations	Early-stage cytotoxicity evaluations indicate acceptable *in vitro* selectivity at active concentrations, but *in vivo* safety and immunotoxicity data are essentially absent	Designed to improve drug-like properties relative to parent natural compounds; however, formulation work is limited to laboratory-scale solutions or simple carriers; no advanced delivery systems or stability-optimized clinical formulations yet.	Development remains fully preclinical; no human studies or clinical registrations have been reported	Duda-Madej et al., 2024
Other synthetic OSC scaffolds (e.g., biphenyl/dibenzofuran sulfur derivatives, hybrid molecules)	Several synthetic OSC derivatives show potent *in vitro* antibacterial activity, including against resistant strains; some also demonstrate antibiofilm effects in phenotypic models	Safety data are mostly limited to *in vitro* cytotoxicity assays; systematic animal toxicity or immunomodulation studies are rare, making human risk assessment premature	Chemically stable and amenable to structural optimization; however, most work remains at the hit/lead stage with no fully developed pharmaceutical formulations or validated stability data under clinical conditions	No clinical trials to date; these compounds should be considered early-stage leads requiring extensive preclinical optimization before any human evaluation	Wang et al. (2022)

### 7.5 Improving OSC bioavailability: delivery system design

One of the major practical barriers to clinical translation of many OSCs is their poor bioavailability—due to instability (e.g., rapid degradation of thiosulfinates), poor aqueous solubility, susceptibility to first-pass metabolism, and limited systemic exposure. However, recent advances in formulation science offer feasible solutions, and several delivery-system approaches have already been proposed and tested for OSCs such as allicin and sulforaphane. For example, a recent comprehensive review on Allicin delivery highlights the use of nanoparticles, liposomes, micelles, gels, and nanoemulsions to improve chemical stability, encapsulation efficiency, and oral bioavailability (Deng et al., 2023). Likewise, for Sulforaphane (SFN)—an isothiocyanate OSC with promising host-directed activity—a recent study demonstrated that microencapsulation using whey- or pea-protein matrices significantly enhanced bioaccessibility during simulated gastrointestinal digestion and improved absorption in a Caco-2/HT29 intestinal cell model compared with unprotected SFN or crude broccoli extract. (Ali Redha et al., 2025) (See Table 5).

**TABLE 5:** Summary table detailing clinical trial parameters (sample size, dosage, outcomes).

Compound	Trial/Citation	Indication	Phase/Design	Sample size	Dose/Formulation	PK/PD biomarkers measured	Primary outcome(s)	Key findings	Limitations
Sulforaphane (SFN)	NCT05084365: Patel P. et al., 2024; NCT05146804: van Steenwijk et al., 2023; Fahey et al., 2025; Saito et al., 2025	Airway antioxidant induction; other indications (varied, e.g., neuro, cancer prevention)	Phase I/II, randomized or open-label (varies by study)	20–100 (varies)	Oral SFN or glucoraphanin + myrosinase; standardized broccoli sprout extracts; doses typically 25–200 µmol SFN-equivalents daily	Plasma SFN and conjugates (LC-MS/MS), urinary mercapturic-acid metabolites; Nrf2 target gene induction (HO-1, NQO1)	PD endpoint: induction of phase II enzymes; safety/tolerability; some disease surrogate endpoints (e.g., Ki-67 in cancer prevention trials)	SFN produces measurable plasma/metabolite exposure and dose-dependent induction of Nrf2 targets; some longer-term trials show potential biomarker or surrogate outcome benefits	Heterogeneity of formulation (isolated SFN vs. glucoraphanin + myrosinase), interindividual conversion variability, and few infection-focused efficacy trials
Aged garlic extract (AGE)/garlic derivatives	NCT03860350: Wlosinska et al., 2021; Wlosinska et al., 2020; Zhao et al., 2024; Rahmatinia et al., 2024	Cardiovascular risk, immunity, metabolic outcomes; limited infection endpoints	Phase II/III (varied), randomized, double-blind, placebo-controlled in many trials	40–200	Capsules standardized to S-allyl-cysteine/AGE; aged garlic extract formulations; dose varies by study	Circulating inflammatory markers (CRP, cytokines), lipid panels	Many trials report modest biomarker improvements (reduced CRP, improved lipids); AGE has been shown to modify immune markers in obesity cohorts	Variable composition among garlic products, limited parent-compound PK for allicin (allicin is labile and rarely detected systemically), and most trials target chronic disease biomarkers rather than infection endpoints	​
Broader SFN trials — registered trials	ClinicalTrials.gov aggregate; review by Saito et al., 2025, and NCT05084365: Patel B. et al., 2024; Yuan et al., 2025	Multiple (oncology prevention, metabolic, neuro, and inflammatory)	Phase 0–II across trials	20–100	Oral SFN or precursor formulations	Urinary mercapturic acids, plasma metabolites, PD gene induction panels	Multiple trials demonstrate safety and PD effects; evidence for clinical efficacy is indication-specific and often preliminary	Few trials target infectious disease endpoints or dual antimicrobial/immunomodulatory outcomes; many are small or surrogate-endpoint focused	​

More broadly, the growing field of nano- and micro-delivery systems for natural bioactives shows that solid lipid nanoparticles, polymeric micelles, nanoemulsions, and self-emulsifying drug delivery systems (SEDDS) can dramatically improve solubility, protect labile compounds from degradation, and promote controlled release and tissue-targeted delivery (Teixé-Roig et al., 2023; Chavda et al., 2022).

Thus, for OSCs to reach their therapeutic potential, rational formulation design should be prioritized early: (I) Use encapsulation (lipid-based carriers, protein matrices, or polymeric nanoparticles) to stabilize reactive OSCs; (II) Employ nanoemulsions or SEDDS platforms to enhance intestinal absorption and bypass first-pass degradation; (III) Design controlled-release or targeted delivery forms (e.g., micelles, liposomes) to optimize tissue distribution and minimize off-target thiol reactivity; (IV) Combine with pro-drug or precursor strategies (e.g., glucosinolate + myrosinase for SFN); when applicable to improve *in vivo* conversion and bioavailability. By integrating these pharmacological strategies, OSCs with otherwise limited systemic exposure may become viable candidates for both antimicrobial and host-directed clinical applications—bridging the gap between *in vitro* promise and *in vivo* reality.

Table 6 summarizes leading delivery strategies that may overcome intrinsic bioavailability and stability limitations of OSCs, highlighting which methods have empirical support and are most amenable to pharmaceutical development.

**TABLE 6:** Delivery strategies to improve OSC bioavailability.

Delivery strategy	Key benefits for OSCs	Representative evidence/Reference
Solid-lipid nanoparticles (SLNs)/lipid-based nanocarriers	Encapsulation of labile, lipophilic OSCs (e.g., allicin) in a lipid matrix provides protection from rapid degradation, improves solubility, facilitates controlled release, and may enhance cellular uptake/tissue distribution	Alyasiri et al. (2023) — “Allicin-loaded solid lipid nanoparticles with chitosan–folic acid coating” demonstrated high encapsulation efficiency (∼86%), controlled release, preserved bioactivity, and reduced toxicity vs. free allicin. Also, Patel B. et al. (2024) review on lipid-based nanoparticles for bioactives outlines general advantages for plant-derived compounds
Protein-based microencapsulation (e.g., whey or plant proteins)	Encapsulating OSCs like isothiocyanates (e.g., sulforaphane) within protein matrices improves chemical stability, protects against degradation during gastrointestinal transit, and enhances bioaccessibility and absorption	Ali Redha et al. (2025) — microencapsulation of broccoli-derived sulforaphane with whey protein improved *in vitro* gastrointestinal bioaccessibility to ∼68% and showed significantly enhanced intestinal absorption in Caco-2/HT29 cell models, compared to unformulated extract. Also, earlier work by Zambrano et al. (2019) reviewed microencapsulation for stabilization of sulforaphane
Nanoemulsions/self-emulsifying drug delivery systems (SEDDS)	For lipophilic OSC derivatives or hydrophobic sulfur compounds, oil-in-water nanoemulsions enhance water dispersibility, protect from hydrolysis, and promote absorption across biological membranes	Choi and McClements (2020) reviewed nanoemulsions for lipophilic nutraceuticals and demonstrated that such systems markedly improve stability, solubility, and bioavailability of unstable or hydrophobic bioactives — a paradigm applicable to lipophilic OSCs. General nano-delivery reviews also highlight their relevance for natural products. (Zakharova et al., 2023)
Organosulfur-based polymers/polymer-backed carriers	Using organosulfur-containing polymers or synthetic carriers allows controlled and targeted release, enhances the stability of reactive sulfur moieties, and can optimize pharmacokinetics	Islam and Zeng (2024) — their review on organosulfur-based polymers outlines design, characterization, and potential of these polymers for controlled delivery and improved biocompatibility of sulfur-based drugs
Microbial or enzyme-prodrug approaches (precursor + activation *in situ*)	For unstable OSCs or isothiocyanates, delivering stable precursors (e.g., glucosinolates) along with activating enzymes (e.g., myrosinase) or engineered microbes may enhance *in vivo* generation and local release at the site — sidestepping instability issues	Li X. et al. (2022) described microbial conversion systems aiming to generate sulforaphane *in situ* *via* engineered gut microbiota, highlighting a plausible “endogenous production” delivery strategy

### 7.6 Standardizing dosing from natural OSC sources: key principles

Natural extracts of OSC-rich botanicals (e.g., garlic) show wide variation in sulfur-compound composition due to factors such as cultivar variety, harvest time, storage, drying/processing method, and extraction protocols (Busia, 2024). This variability undermines reproducibility, complicates dose definition, and makes pharmacokinetic/pharmacodynamic (PK/PD) matching difficult for clinical use.

To overcome these challenges, the following steps should be adopted: (I). *Quantitative standardization of active moieties*—Rather than use “garlic extract” as a bulk ingredient, preparations must be analytically characterized (e.g., by HPLC, LC–MS/MS) to quantify key OSCs (such as allicin or stable disulfides) or validated surrogate biomarkers (e.g., metabolites). Only batches meeting defined content thresholds should be accepted for clinical formulation. This ensures batch-to-batch consistency and allows accurate dosing (Shirke et al., 2014). (II). Use of well-defined extract formats (e.g., aged garlic extract, standardized powder, or purified OSC fraction) — As shown in pharmacokinetic studies, formulations with known allicin bioavailability led to more predictable exposure than raw garlic or uncharacterized extracts (Lawson and Hunsaker, 2018). (III). Employ pharmaceutic-grade extraction and manufacturing under GMP conditions with quality-control protocols (identity, purity, content, stability, absence of contaminants) in line with herbal-drug standardization guidance—tasks long recognized as essential when moving from nutraceutical use to clinical therapeutic development (Chaachouay and Zidane, 2024). (IV). Use of PK/PD biomarkers to define effective dose ranges—Because direct measurement of OSC in tissues may be challenging, surrogate markers (e.g., downstream redox- or thiol-modification biomarkers, mercapturic-acid conjugates, Nrf2 target-gene induction) should be used to calibrate individualized or population-level dosing. This shifts dosage control from “mg of garlic” to “biologically effective dose.”

By combining rigorous chemical standardization, GMP-like manufacturing, and biomarker-based PK/PD calibration, it is feasible to overcome natural-source variability and define reproducible, clinically meaningful OSC doses—transforming OSCs from variable botanicals into pharmaceutical-grade agents**.**

## 8 Clinical evidence (human and preclinical)

This section summarizes verifiable human and preclinical evidence for organosulfur compounds (OSCs) relevant to their dual antimicrobial–immunomodulatory potential. We focus on (i) OSCs with verifiable human PK/PD or biomarker data, (ii) robust preclinical infection and immunomodulation models, (iii) gaps and limitations that constrain interpretation, and (iv) practical next steps for evidence-driven clinical development.

### 8.1 Verified human evidence (select OSCs)

*Sulforaphane (SFN, isothiocyanate class)* - Sulforaphane has the most extensive, verifiable human data among OSCs. Controlled supplementation and feeding studies have quantified SFN and its mercapturic-acid pathway metabolites (SFN–GSH, SFN–Cys, SFN–NAC) in plasma and urine using validated LC–MS/MS methods, enabling reliable PK characterization and PD linkage to Nrf2 target gene induction (HO-1, NQO1) in peripheral blood cells. Human trials and controlled interventions have reported dose-dependent induction of Nrf2-responsive transcripts and some reductions in selected inflammatory biomarkers, supporting SFN as a tractable OSC for human proof-of-mechanism studies. Importantly, PK is influenced by precursor form (glucoraphanin vs. purified SFN), myrosinase activity, and inter-individual microbiome differences, all of which must be controlled or measured in clinical protocols (Lu, 2024; Ma et al., 2023). As summarized in Table 4, sulforaphane remains the only OSC class with human-level PK/PD data supportive of early-phase translation.”

*Aged garlic extract/allicin-derived supplements* - Human studies of garlic preparations and aged garlic extract document measurable downstream volatile metabolites (e.g., allyl methyl sulfide) and varying biomarker responses. However, intact allicin is rarely detected in systemic circulation after oral administration because of rapid chemical transformation; as a consequence, human PK data for parent allicin are limited and highly formulation-dependent.

Systematic reviews emphasize wide variability in bioavailability across preparations and call for standardized analytical methods and well-characterized formulations when planning translational studies. Thus, while there are human data supporting biological activity for garlic preparations, direct evidence for intact allicin systemic exposure is weak and must be interpreted cautiously (Nakamoto et al., 2025; Tedeschi et al., 2022).

*Other OSCs -* For most other OSCs (ajoene, DADS, and synthetic stabilized OSCs), human pharmacokinetic or controlled biomarker data are either limited or absent in the peer-reviewed literature (2020–2025). Accordingly, these compounds remain at preclinical or early-translational status pending validated PK/PD studies (Oluwabusola et al., 2022; El-Saadony et al., 2024).

### 8.2 Preclinical efficacy with translational relevance

*Quorum-sensing and anti-virulence effects (ajoene, ajoene analogues) -* Ajoene—isolated from garlic—has robust *in vitro* evidence of quorum-sensing inhibition in *Pseudomonas aeruginosa*, with genome-wide transcriptional changes in QS-regulated virulence factors and demonstrable biofilm inhibition; *in vivo* mouse pulmonary infection models treated with ajoene showed reduced bacterial burden and synergistic activity with tobramycin in biofilm-associated settings. These studies provide mechanistic proof-of-concept for anti-virulence strategies based on sulfur chemistry (Jakobsen et al., 2012; Oluwabusola et al., 2022).

*Antimicrobial and immunomodulatory proof-of-concept (allicin-type interventions) -* Preclinical studies demonstrate that allicin and allicin-enriched formulations reduce pathogen load in topical and mucosal infection models and attenuate inflammatory damage (reduced neutrophilic infiltration, lower cytokine levels) in rodent challenge models. These data support the possibility of local-delivery applications (e.g., topical, inhalation, or GI mucosal targeting) where the short-lived reactive parent compound can act at the site of infection without requiring prolonged systemic exposure (Oluwabusola et al., 2022; Tedeschi et al., 2022).

*Synthetic OSC derivatives and efflux/anti-biofilm activity* **-** Recent medicinal chemistry work on stabilized sulfonyl and sulfoxide analogues shows promising *in vitro* potency against multidrug-resistant strains, with activity against efflux systems and biofilms *in vitro* and in select animal models. These findings highlight a translational path that leverages synthetic chemistry to retain sulfur-dependent mechanisms while improving stability and PK properties; however, these are preclinical data and require ADME/toxicity validation before human testing (Oluwabusola et al., 2022; Chadha et al., 2022).

### 8.3 Deficits, limits, and transparency about evidence gaps

*No confirmed clinical efficacy trials for infectious indications -* Despite promising preclinical data, as of the 2020–2025 literature window (Guillamón et al., 2021), there are no PubMed-indexed Phase II or larger randomized clinical trials demonstrating that any OSC reduces infection-related clinical endpoints (e.g., mortality, time to clearance, hospitalization) when used as an antimicrobial adjunct. Clinical trial registries list biomarker and safety studies (not efficacy trials) for SFN and dietary glucoraphanin preparations (NCT05146804: van Steenwijk et al., 2023), but completed efficacy trials for infectious disease are lacking; this important absence must be stated explicitly in any translational narrative.

*PK challenge for labile OSCs -* Allicin-class OSCs’ rapid reactivity and decomposition mean parent-compound systemic exposure is usually undetectable after oral dosing (Nakamoto et al., 2025); thus, establishing systemic PK/PD is challenging without chemical stabilization or targeted delivery (topical/inhalation/GI). As a result, claims of systemic dual-action effects for these compounds in humans may be elusive until validated, chemically stabilized formulations or targeted delivery are available.

*Heterogeneity of botanical preparations and assay methods -* Studies using “garlic extracts,” “aged garlic,” or unstandardized botanical material vary widely in precursor content (alliin/glucoraphanin), myrosinase activity, and analytical methods, creating heterogeneity that hampers cross-study comparisons. For isothiocyanates, uncontrolled dietary matrix and microbiome effects introduce additional variability that must be measured in human trials. Systematic standardization of source material and validated bioanalytical assays are prerequisites for reproducible clinical studies (Nakamoto et al., 2025).

### 8.4 Recommended evidence-focused next steps for clinical development

Below are pragmatic, evidence-aligned steps to advance OSCs from mechanistic promise toward human proof-of-concept, emphasizing verifiable endpoints and risk mitigation:

- *Prioritize OSCs with measurable human PK/PD (e.g., sulforaphane)* **-** Use SFN as a clinical test case to establish PD endpoints (Nrf2-target induction, cytokine panels) and to refine assay timing and sampling windows (0.5–8 h post-dose for plasma metabolites; 24–48 h for urinary mercapturic metabolites). Early human SAD/MAD studies should measure both exposure and molecular PD markers (Lu, 2024).
- *Develop validated analytical methods and standardized formulations* **-** For each candidate, create stability-indicating assays for parent compound and major metabolites (LC–MS/MS), and produce standardized formulations (e.g., defined glucoraphanin + myrosinase activity; stabilized allicin formulations). This enables reproducible PK/PD bridging (Nakamoto et al., 2025).
- *Design short, biomarker-driven human proof-of-mechanism studies before efficacy trials -* Use *ex vivo* challenge assays, controlled human infection models (where ethically feasible), or surrogate endpoints (mucosal cytokine responses, phagocyte function assays) to test dual-action hypotheses and to identify minimally effective exposures. Emphasize safety, especially thiol-reactivity–related off-target risks (Lu, 2024; Dinkova-Kostova and Copple, 2023).
- *Targeted local-delivery strategies for labile OSCs -* For allicin-type OSCs, prioritize topical, inhalation, or GI-targeted formulations with localized PK/PD readouts to exploit local antimicrobial and immunomodulatory effects while avoiding systemic instability. Preclinical infection models should demonstrate local target engagement and safety before human testing (Oluwabusola et al., 2022; Tedeschi et al., 2022).
- *Preclinical safety and off-target profiling -* Because OSCs can react with host thiols, conduct comprehensive off-target profiling (proteomics of S-thiolation in host tissues), dose–response cytotoxicity panels, and ADME/tox studies before first-in-human studies. Use these data to establish safe starting doses and monitoring plans (Dinkova-Kostova and Copple, 2023; Nakamoto et al., 2025).

## 9 One Health: relevance of organosulfur compounds (OSCs) to cross-sectoral antimicrobial ecology

**FIGURE 7:**
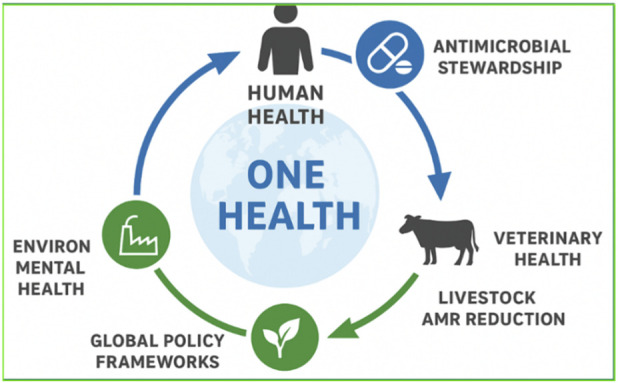
OSCs as a One Health bridge across human, veterinary, and environmental health.

One Health frames antimicrobial resistance (AMR) as an ecological problem spanning human, animal, and environmental health. Within this framework, organosulfur compounds (OSCs) have clear, evidence-backed intersections with each domain that merit concise, mechanistic discussion rather than speculative extrapolation. First, OSCs influence microbial ecology in soils and plants: sulfur-containing phytochemicals shape rhizosphere communities, influence pathogen suppression, and modulate microbial sulfur metabolism—processes that ultimately affect zoonotic reservoirs and agricultural pathogen dynamics. Second, OSCs used in animal feed or crop protection can alter microbiome composition and antimicrobial-selection pressures in livestock and agricultural soils. Third, plant-derived OSCs and their metabolites enter environmental compartments (soil, water) where abiotic and biotic transformations alter chemical speciation and biological activity, with implications for environmental persistence and selection on the resistome. These cross-sectoral interactions position OSCs as relevant agents to consider within One Health strategies, but evidence supports cautious, evidence-driven engagement rather than broad claims of ecosystem-level benefit (Figure 7).

Evidence indicates that OSCs can potentiate antibiotic activity by mechanisms including efflux pump inhibition, biofilm disruption, and cell membrane destabilization, thereby restoring antibiotic susceptibility in multidrug-resistant (MDR) pathogens. For example, allicin has been shown to synergize with β-lactams against *Staphylococcus aureus* and *Pseudomonas aeruginosa*, thus calling for further animal experiments to validate *in vitro* synergy (Choo et al., 2020). Additionally, Parallel investigations (Bianchi M et al., 2024) highlight porin-mediated uptake pathways as viable targets for resistance reversal. Importantly, such adjuvant effects may allow for dose sparing, reducing selective pressure for resistance while minimizing toxicity risks in patients. These features directly support antimicrobial stewardship goals, where optimization of therapy involves not only judicious use of antibiotics but also the incorporation of safe adjuncts to preserve efficacy.

Mechanistically, OSCs’ impact on One Health centers on sulfur redox chemistry and microbial thiol metabolism. Sulfur compounds can perturb bacterial redox homeostasis and signaling (including quorum sensing), reduce virulence factor expression in key pathogens, and influence soil microbial community composition in ways that may lower pathogen load or alter resistome composition under controlled conditions. For example, ajoene (a garlic-derived OSC) demonstrates quorum-sensing inhibition in *Pseudomonas aeruginosa* and modulates virulence expression *in vitro* and in preclinical biofilm models; such anti-virulence action has implications for both clinical and agricultural control of biofilm-associated pathogens.

However, the ecological consequences of applying OSCs at agricultural scales remain understudied: field-level trials, longitudinal resistome analyses, and assessments of non-target effects are required before recommending OSCs for large-scale One Health interventions (Gerson and Hinckley, 2023; Hermes et al., 2023).

### 9.1 Organosulfur compounds (OSCs) in livestock and aquaculture systems

In livestock production, evidence from poultry and swine nutrition shows that Allium-derived OSCs, mainly from garlic, are actively being evaluated as alternatives or adjuncts to in-feed antibiotics. Recent reviews report that garlic-based additives improve growth performance, gut health, antioxidant status, and immune markers in broilers and layers, largely attributed to sulfur-containing phytochemicals such as allicin, S-allyl cysteine, and related organosulfur compounds (Abd El-Ghany, 2024; Guillamón et al., 2021). In pigs, Allium spp. Extracts supplemented in feed improved weight gain and feed conversion and were associated with favorable shifts in gut microbiota and short-chain fatty acid metabolism, supporting a combined antimicrobial and microbiome-modulating effect *in vivo* (Sánchez et al., 2020). More recent broiler trials indicate that garlic powder can enhance growth and carcass traits without adverse effects on liver or kidney function, reinforcing its feasibility as a relatively safe phytogenic additive under commercial-like conditions (Djamen et al., 2024). Sulforaphane (SFN) is also beginning to appear in livestock research. Experimental studies in broiler chickens suggest that dietary SFN or SFN-rich preparations can modulate immune and oxidative-stress pathways, including Nrf2/NF-κB signalling, and may counteract immunosuppression induced by aflatoxin B1 exposure (Suleyman, 2025). Although these data are preliminary and largely confined to controlled experimental settings, they demonstrate that OSCs are not only conceptually but also practically deployable as host-directed and microbiota-modulating agents in animal production systems seeking to reduce antibiotic inputs.

In aquaculture, garlic-based OSC sources are being actively tested as immunostimulants and disease-mitigating feed additives. Experimental work in finfish and shellfish shows that dietary garlic extract can improve growth, survival, hematological parameters, and non-specific immune responses, while enhancing resistance to bacterial pathogens (Ismarica et al., 2025; Rahul Sandeep et al., 2025). For example, garlic extract supplementation in pomfret or catfish fry has been reported to increase survival and improve blood immune profiles, and garlic powder or extract has reduced Vibrio-associated mortality in shrimp by leveraging allicin-mediated antibacterial activity (Ismarica et al., 2025; Delgado et al., 2023). A recent systematic review of phytobiotics in finfish and shellfish highlights Allium-based preparations as among the most consistently beneficial plant additives, with effects spanning growth promotion, immune enhancement, and improved disease resistance (Rahul Sandeep et al., 2025; Kolygas et al., 2025).

From a One Health perspective, these data suggest both opportunity and responsibility: OSCs could help reduce reliance on conventional antibiotics in animal production, but their wider deployment should be accompanied by rigorous assessment of environmental fate, resistome impacts, and food residue safety to avoid simply replacing one poorly understood selection pressure with another.

From a policy perspective, integrating OSC-based interventions into livestock systems must align with One Health governance principles that balance animal productivity, human health, and environmental protection. While OSCs show promise as antibiotic-sparing agents, their deployment should happen within regulated frameworks that require (i) standardized feed-grade OSC formulations with verified chemical profiles, (ii) routine monitoring of microbial communities and AMR gene levels in farm environments, and (iii) transparent reporting of on-farm effectiveness and ecological impacts. Coordinated guidelines—similar to those used for antibiotic alternatives and feed additives—would ensure that OSC adoption does not unintentionally create selection pressures affecting human clinical AMR or disrupting soil and aquatic microbiomes. Incorporating OSC use into integrated surveillance systems and best agricultural practices is therefore a key policy approach to maximize their dual benefits while protecting public health and ecological stability.

### 9.2 OSCs in wastewater and environmental microbial ecosystems

#### 9.2.1 OSCs and antimicrobial resistance in wastewater

Because OSCs exert anti-virulence pressure and thiol-mediated stress rather than classic target-specific killing, interest is emerging in their potential to reduce antimicrobial resistance (AMR) selection in wastewater systems (Cruz et al., 2024; Li et al., 2025). Laboratory microcosm studies demonstrate that garlic-derived OSCs (e.g., thiosulfinates and polysulfides) can suppress growth and quorum-regulated biofilm development of wastewater-relevant pathogens, including *Pseudomonas* (Li X. et al., 2020 *)*, *Enterobacterales* and *Acinetobacter* species (Corbu et al., 2021; Bhatwalkar et al., 2021), at low-to-mid micromolar exposures—conditions consistent with expected environmental dilution profiles. By disarming pathogens and decreasing plasmid-encoded virulence expression, OSCs may lower horizontal gene transfer efficiency and thus indirectly reduce AMR propagation in water systems (Qiu et al., 2024); however, there is no direct study on OSCs (such as garlic-derived thiosulfinates or polysulfides) specifically lowering HGT in water systems. But their anti-biofilm and quorum-sensing inhibition properties suggest a plausible indirect effect (Michaelis and Grohmann, 2023). These mechanisms complement the One Health framework advanced in this review by linking direct antimicrobial activity to environmental AMR stewardship.

#### 9.2.2 Ecological and toxicity considerations

However, OSC deployment in wastewater environments must consider redox reactivity and potential ecological toxicity. Highly electrophilic OSCs (e.g., allicin-class thiosulfinates) can non-selectively modify thiol-dependent proteins in non-target aquatic microbiota, potentially altering soil and water microbial community structure (Liang et al., 2025; Müller et al., 2016). Isothiocyanates and synthetic OSC derivatives are more chemically stable and could persist longer, raising concerns regarding selective pressure on environmental resistomes if concentrations exceed ecological no-effect thresholds (Hu et al., 2025; Song et al., 2025)Accordingly, any proposals for OSC use in wastewater remediation should incorporate environmental-fate tracking, biodegradation studies, and resistome monitoring to ensure that AMR suppression benefits are not offset by unintended ecological disruption.

#### 9.2.3 Regulatory considerations

The integration of OSCs into stewardship and One Health frameworks requires regulatory, economic, and policy alignment. Several OSC-rich plant extracts are already classified as Generally Recognized as Safe (GRAS) by the U.S. FDA, facilitating their adoption as feed additives or nutraceuticals (Martins et al., 2016). However, standardized manufacturing, quality control, and clinical validation remain essential for their wider deployment in healthcare. Regulatory agencies such as the EMA and FDA are increasingly open to phytochemical-derived adjuvants as long as rigorous preclinical and clinical evidence support efficacy and safety. At the policy level, incorporation of OSC-based strategies could strengthen global AMR action plans, especially in low- and middle-income countries where access to last-line antibiotics is limited. By bridging human, animal, and environmental health applications, OSCs exemplify the translational ethos of One Health, supporting not only therapeutic innovation but also sustainable AMR containment.

For a large-scale production, scaling up OSC production will require proactive stewardship to protect environmental microbiomes and limit resistome selection pressures. Sustainable agricultural sourcing from Brassica and Allium crops should prioritize crop rotation and soil nutrient management to avoid ecosystem disruption, while purification and processing steps should incorporate sulfur-waste recycling to prevent contamination of shared water systems at the human–animal–environment interface. Embedding environmental fate studies and resistome monitoring into early development pipelines will ensure OSC-based interventions evolve in alignment with One Health principles and antimicrobial stewardship goals.

Practical One Health considerations for OSC development include: *(I). Targeted, local use over blanket application -* Given the potential for off-target ecological effects, prioritize localized OSC applications (e.g., topical, post-harvest surface treatment, targeted livestock interventions) with monitoring of soil and gut microbiomes (Bouranis and Chorianopoulou, 2023; Çakilli et al., 2025). *(II). Environmental fate and transformation studies -* Characterize abiotic degradation pathways and microbial biotransformation of OSCs in soil and water; such data are needed to predict persistence, metabolite formation, and potential impacts on the environmental resistome (Ottosen et al., 2024; Vigneron et al., 2021; Tang, 2020). *(III). Integrated monitoring -* Any translational pathway that involves agricultural or veterinary uses should include longitudinal resistome surveillance (metagenomics) and functional assays to measure selection pressure (Keenum et al., 2022; Napit et al., 2025; Apjok et al., 2023).

**FIGURE 8:**
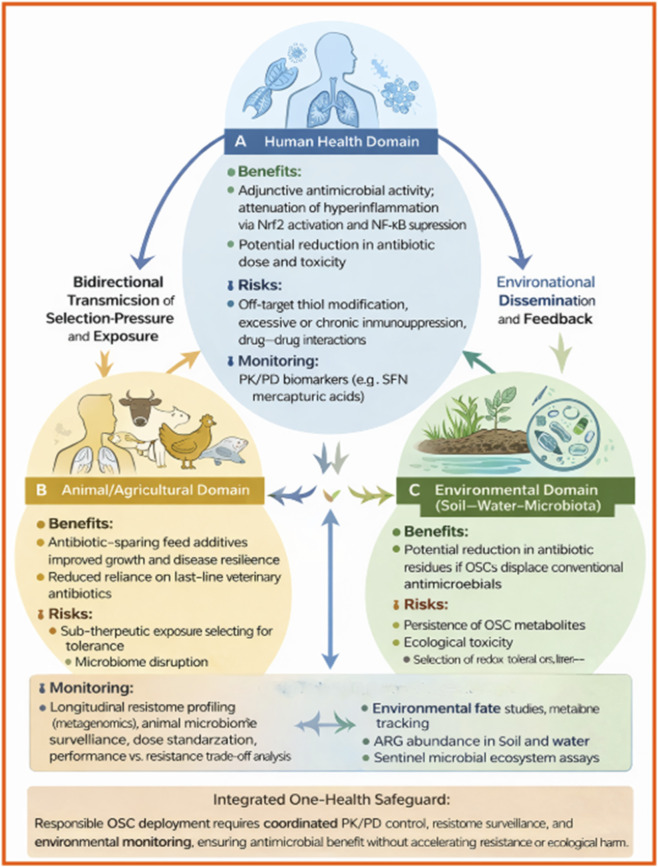
One-Health Schematic for OSCs - Benefits, Risks, Monitoring requirements Across Human-Animal -Environment Interface.

Figure 8 illustrates the One Health framework for organosulfur compounds (OSCs), summarizing the interconnected benefits, risks, and monitoring requirements across human, animal/agricultural, and environmental domains that underpin their responsible translational deployment.

## 10 Future perspectives

**FIGURE 9:**
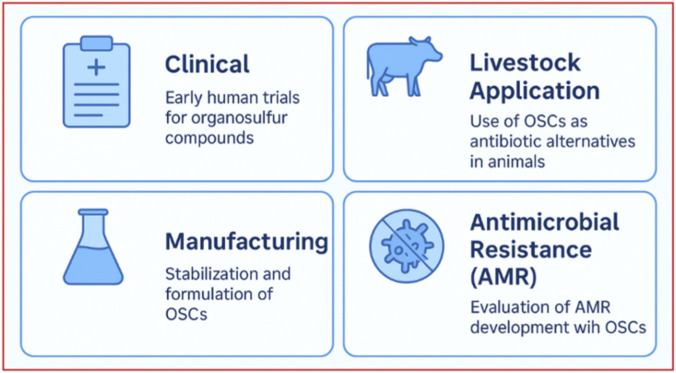
Key future perspectives for advancing organosulfur compounds (OSCs) - The infographic outlines four priority areas for OSC development: (1) Clinical–establishing pharmacokinetics and early human proof-of-mechanism; (2) Livestock Application–evaluating OSCs as antibiotic alternatives in animal production; (3) Manufacturing–improving stabilization and scalable formulation; and (4) AMR–assessing how OSC use may influence antimicrobial-resistance dynamics across One Health settings.

This section outlines the key plausible perspectives required to advance organosulfur compounds (OSCs) from mechanistic promise to translational, agricultural, and One Health applications (Figure 9).

### 10.1 Clinical

From a clinical translation perspective, dual-action OSCs should be viewed as potential adjuncts to, rather than replacements for, contemporary last-line antibiotic regimens. Although ceftazidime/avibactam-based therapy has improved outcomes in carbapenem-resistant Gram-negative infections, real-world cohorts still show incomplete microbiological clearance and non-trivial 30-day mortality, even under optimized dosing conditions (Zhuang et al., 2023). Large-scale syntheses of clinical data on multidrug-resistant Gram-negative infections similarly conclude that new BL/BLI combinations and cefiderocol have shifted—but not eliminated—the high-risk nature of these infections, and that resistance to these agents is already being reported (Macesic et al., 2025; Liu et al., 2025). In this context, OSCs are best conceptualized as host- and pathogen-directed co-therapeutics that may (i) lower pathogen burden through redox-mediated and membrane-active mechanisms while (ii) re-balancing dysregulated inflammatory and oxidative responses, thereby complementing last-line antibiotics rather than competing with them. Clinical outcome data with ceftazidime/avibactam and other novel agents reinforce that further gains will likely come from rational adjunctive strategies; OSCs, if rigorously evaluated, may occupy such a niche as dual-action, host-pathogen-modulating partners to existing last-line antibiotics (Macesic et al., 2025; Zhuang et al., 2023).

### 10.2 Livestock application

The potential for OSCs to serve as antibiotic-sparing agents in livestock production has gained increasing attention within the One Health framework. In poultry, swine, and aquaculture, OSC-rich Allium and Brassica extracts have been shown to reduce pathogen load, improve intestinal barrier integrity, modulate inflammatory responses, and enhance feed efficiency—effects that map directly onto the dual-action paradigm described earlier (Abd El-Ghany, 2024). Recent trials demonstrate that OSCs such as allicin, diallyl disulfide, and sulforaphane analogues can suppress *Salmonella*, *Campylobacter*, and *Clostridium* proliferation while concurrently attenuating gut inflammation and oxidative stress markers, leading to improved animal performance under commercial-like conditions (Makuch et al., 2025; Lu et al., 2012; Tang et al., 2021).

Their mechanisms in livestock largely parallel those observed in human or murine systems—thiol modification of microbial proteins, interference with quorum sensing, and Nrf2-associated mucosal protection (Li et al., 2021; Bedir et al., 2025; Gruhlke et al., 2019)—suggesting conserved biological targets across host species.

Despite these promising outcomes, several translational challenges must be addressed before OSCs can be positioned as reliable antimicrobial alternatives in animal agriculture. The chemical instability of many natural OSCs, particularly thiosulfinates such as allicin, complicates dosing accuracy in feed matrices and can generate variable bioactive profiles depending on storage, processing, and environmental conditions (Kothari et al., 2019). Additionally, livestock metabolism and rumen/gut microbiota may transform OSCs into secondary sulfur metabolites with distinct, and not always predictable, biological effects (Yeoman et al., 2021). Therefore, standardized feed formulations, controlled-release encapsulation strategies, and dose–response studies across species remain essential. Equally critical is the implementation of resistome surveillance in farms adopting OSC interventions, as even non-antibiotic antimicrobial pressures can shape microbial community structure and AMR gene dissemination. Integrating OSCs into livestock health programs thus requires both mechanistic justification and rigorous environmental monitoring to ensure that their dual-action benefits do not inadvertently contribute to unintended ecological selection pressures.

### 10.3 Manufacturing

From a manufacturing perspective, several OSC subclasses are technically feasible for large-scale production. Sulforaphane and its glucosinolate precursor are already produced at an industrial scale from standardized Brassica biomass for dietary supplements and clinical research (González et al., 2021; Fahey et al., 2015; Bell and Wagstaff, 2017), demonstrating that controlled extraction and formulation of isothiocyanates is achievable. In contrast, highly labile thiosulfinates such as allicin, although abundant in garlic and potent *in vitro*, pose major challenges for API-grade production due to rapid decomposition (Zhou et al., 2025; Borlinghaus et al., 2021). This instability poses major challenges for its development as an API-grade (active pharmaceutical ingredient) drug, restricting its use mainly to complex garlic preparations rather than as a purified pharmaceutical (Zhou et al., 2025; Borlinghaus et al., 2021). Synthetic OSC derivatives and hybrids can, in principle, be manufactured using established organic synthesis (Sead et al., 2025; Hanek and Żak, 2024) or microbial fermentation routes (Gong et al., 2020; Luo et al., 2023), but these remain at the preclinical stage and have not yet progressed to GMP-scale, regulatory-oriented production (Robinson et al., 2020). Overall, large-scale production is feasible for selected OSCs, but only those with adequate chemical stability and standardizable process control—such as sulforaphane—are realistically positioned for near-term clinical development. Large-scale OSC production should also incorporate One Health considerations, including sustainable sourcing of Brassica and Allium biomass to avoid monoculture-driven soil nutrient depletion and microbial imbalance. Additionally, closed-loop extraction and waste-valorization systems may help limit sulfur-rich effluent release, preventing unintended impacts on environmental microbiomes and resistome dynamics.

### 10.4 Antimicrobial resistance (AMR) development

While organosulfur compounds (OSCs) such as garlic-derived thiosulfinates and other phytochemicals have shown broad-spectrum antimicrobial activity and appear less prone to resistance development than many single-target antibiotics, the possibility of adaptive bacterial resistance to OSCs cannot be excluded. Indeed, a recent review explicitly notes that bacteria exposed to plant-derived phytochemicals—including OSCs—may evolve resistance through general stress-response mechanisms such as efflux pump upregulation, enzymatic detoxification, or metabolic remodeling under sublethal exposures (Vaou et al., 2021). To rigorously evaluate this risk, we recommend the following studies: *Serial-passaging experiments under sub-inhibitory OSC concentrations:* repeated exposure of representative pathogens (Gram-positive and Gram-negative) to sub-MIC OSC levels over many generations. Monitor for incremental increases in MIC values, biofilm formation capacity, efflux pump expression, and stress-response genes. *Genomic and transcriptomic analyses of adapted strains*: assess whether resistance is mediated by mutations, horizontal gene transfer, or upregulated protective pathways (e.g., antioxidant enzymes, efflux systems). *Cross-resistance testing: determine* whether adaptation to OSCs confers cross-resistance to conventional antibiotics or other stressors, which would have serious implications for antimicrobial stewardship. *Environmental and ecological surveillance:* if OSCs are deployed widely (e.g., in agriculture, wastewater), monitor microbial communities and resistome composition to ensure no unintended selection for more resilient, multi-stress-tolerant pathogens. Until such data are available, claims that OSCs are “resistance-proof” must remain guarded. These research investments will help clarify whether OSCs can deliver on their promise as sustainable, dual-action therapeutics—or whether their use necessitates the same cycle of vigilance required for conventional antibiotics.

### 10.5 Synthetic biology approaches: enabling tools for OSC stabilization, production, and mechanistic resolution

Synthetic biology offers practical and experimentally validated strategies for addressing several longstanding challenges in OSC research—particularly instability, inconsistent precursor content, and limited access to structurally defined analogues. Rather than positioning synthetic biology as a translational solution itself, it should be framed as an enabling platform that produces the *molecular tools* required for rigorous pharmacological evaluation.

*Microbial biosynthesis of stabilized OSC analogues -* Engineering microbial chassis such as *Escherichia coli* or *Saccharomyces cerevisiae* to overexpress cysteine desulfurases, γ-glutamyltranspeptidases, or myrosinase-like enzymes enables controlled biosynthesis of sulfoxides, isothiocyanates, and sulfonyl derivatives with greater stability than their native plant precursors (Jara et al., 2021). Such approaches have been used to improve yields and purity of sulfur-based phytochemicals, allowing for downstream pharmacological testing with reduced batch-to-batch variability (Sadanov et al., 2025; Kumadoh et al., 2023). This strategy is particularly applicable to sulforaphane-class compounds, whose natural conversion from glucoraphanin depends heavily on plant-processing variables and human gut microbiota.

*Pathway refactoring to explore structure–activity relationships* **-** Synthetic biology allows modular reconstruction and modification of OSC biosynthetic pathways to generate libraries of analogues differing in electrophilicity, lipophilicity, or sulfur oxidation state (Schuergers et al., 2017). These analogue libraries provide critical structure–activity insights into antimicrobial potency, redox reactivity, and off-target thiol modification patterns—data that cannot be reliably captured from heterogeneous botanical extracts.

*Testing hypotheses of OSC thiol reactivity and virulence modulation* - Engineered strains with altered thiol-redox capacity (e.g., glutathione-deficient mutants, Nrf2 or Keap1 variants) provide controlled systems to dissect the mechanistic contribution of sulfur electrophilicity, S-thiolation, and redox stress responses to OSC-mediated antimicrobial or immunomodulatory activity (Yamamoto et al., 2018). This approach aligns synthetic biology directly with mechanistic validation rather than speculative therapeutic claims.

*Overall*, synthetic biology supports OSC research by enabling precise molecular production, pathway-level manipulation, and controlled testing systems that address instability, variability, and mechanistic uncertainty—the core translational barriers highlighted.

### 10.6 Multi-omics applications: resolving mechanisms, measuring off-target effects, and monitoring One Health impacts

Recent advances in genomics have greatly expanded the scope of bioprospecting, enabling systematic identification of bioactive natural compounds derived from microorganisms and plants (Chigozie et al., 2025b). Multi-omics tools—proteomics, metabolomics, transcriptomics, and metagenomics—provide empirical datasets essential for deconvoluting OSC mechanisms and characterizing their biological impact across human, animal, and environmental systems. Their role is not to propose new OSC applications but to generate the mechanistic and safety evidence needed for responsible translation.

*Proteomics for mapping on- and off-target S-thiolation* **-** OSCs can covalently modify protein thiols, but the breadth and functional consequences of these modifications remain under-characterized. Redox-proteomics platforms now map S-thiolation and sulfenylation events with high positional accuracy, enabling identification of microbial targets (e.g., redox-sensitive transcription factors) and potential host off-targets (Sheehan and McDonagh, 2020). These data are critical for defining therapeutic windows and mitigating toxicity risks.

*Metabolomics for characterizing OSC biotransformation -* Sulfur metabolites differ dramatically across matrices (plasma vs. gut vs. soil). LC–MS/MS metabolomics has become the gold standard for identifying OSC-derived conjugates (GSH, cysteine, N-acetylcysteine adducts), mapping degradation pathways, and quantifying exposure–response relationships—for example, the mercapturic-acid pathway metabolites of sulforaphane that serve as validated pharmacokinetic markers in humans (Langston-Cox et al., 2020).

*Transcriptomics for mechanistic immune and stress-response profiling - RNA*-seq studies in immune cells and epithelial tissues reveal consistent transcriptional signatures associated with OSC exposure—particularly activation of Nrf2-driven antioxidant pathways and suppression of NF-κB inflammatory cascades (Gao et al., 2022; Meng et al., 2017). Such data support mechanistic hypotheses but also inform safety evaluation by identifying stress-response genes activated at higher doses.

### 10.7 Metagenomics for monitoring one health impacts on microbial communities and resistome

Metagenomics has become a powerful genomic tool that has made possible the sequencing of genetic material from entire microbial communities without the need for culturing individual organisms (Chigozie VU. et al., 2025). Given that OSCs influence microbial redox environments, quorum sensing, and ecological competition, metagenomic surveillance in soil, water, and livestock microbiomes is essential for assessing unintended selection pressures. Several One Health AMR studies emphasize the importance of high-resolution resistome profiling to detect sub-lethal selection, mobile genetic element transfer, or shifts in pathogen abundance following exposure to phytochemicals or redox-active natural products (Waskito et al., 2022). By integrating these multi-omics platforms, OSC research can move from descriptive observations to quantifiable mechanistic models, ensuring translational decisions are based on robust, multi-layered biological evidence.

## 11 Limitations and translational barriers

Despite substantial mechanistic and preclinical progress, the translation of organosulfur compounds (OSCs) from experimental systems to clinical or One Health applications remains constrained by several interrelated limitations that warrant careful consideration. Foremost among these is chemical instability, particularly for thiosulfinates such as allicin, which rapidly decompose and undergo nonspecific thiol reactions under physiological conditions. This lability complicates accurate quantification, reproducible exposure, and systemic delivery, thereby limiting their suitability as conventional drug candidates. While analytical advances and stabilization strategies have improved the assessment of certain OSCs—most notably sulforaphane metabolites—parent-compound instability for allicin-like molecules remains a central barrier, necessitating a focus on measurable metabolites, localized delivery, or more stable synthetic analogues.

A second major challenge is pharmacokinetic variability and microbiome dependence. Human exposure to isothiocyanates such as sulforaphane is strongly influenced by dietary matrix, enzymatic conversion, and inter-individual differences in gut microbial activity, leading to wide variability in systemic and tissue concentrations. Without stratification or biomarker-guided dosing, this variability complicates dose–response interpretation and trial design. These issues underscore the need for early incorporation of validated exposure markers (e.g., mercapturic acid conjugates) and consideration of microbiome covariates in translational studies.

Relatedly, the risk of off-target thiol reactivity presents an important safety consideration. The same electrophilic sulfur chemistry that enables covalent targeting of microbial proteins may also modify host proteins if exposure is excessive or poorly controlled. Proteomic studies have begun to map such off-target interactions, but systematic integration of redox- and activity-based proteomics into development pipelines is still limited. Defining therapeutic windows will therefore require rigorous dose titration, off-target profiling, and functional toxicity assays prior to human application.

Another critical limitation is the absence of rigorous clinical efficacy data for infectious indications. To date, no adequately powered randomized trials have demonstrated that OSCs improve infection-related outcomes, either alone or as antibiotic adjuncts. Human studies have largely focused on pharmacokinetics and pathway-level pharmacodynamic readouts rather than clinical endpoints such as pathogen clearance or reduced antibiotic use. While this gap precludes claims of therapeutic readiness, the consistency of antimicrobial, anti-virulence, and host-modulatory effects across diverse preclinical models provides a strong rationale for biomarker-driven early-phase studies designed explicitly around infectious contexts.

Compounding this issue is the lack of validated pharmacodynamic biomarkers that directly link OSC exposure to antimicrobial efficacy in humans. Although sulforaphane represents a notable exception—with reproducible exposure metrics and pathway-linked host markers—most OSC subclasses lack biomarkers that integrate microbial and host responses. Emerging evidence suggests that OSC activity may occur at sub-MIC levels through virulence attenuation or redox modulation, highlighting the need for composite PD panels that combine microbial endpoints (e.g., biofilm or quorum-sensing suppression) with host inflammatory or redox markers. Such frameworks remain under development and require formal qualification.

The context- and dose-dependence of immunomodulatory effects further complicates translation. OSCs can attenuate damaging inflammation in hyperinflammatory states, yet excessive or prolonged modulation may impair host defense, particularly in immunocompromised settings. Most data derive from controlled experimental models with limited relevance to heterogeneous patient populations, emphasizing the need for biomarker-guided dosing, short-course or localized exposure, and infection-relevant models that assess both microbial clearance and immune competence.

Finally, One Health considerations—including environmental persistence, ecological selection, and resistome impacts—are supported by limited empirical data. While OSCs are generally reactive and may be less persistent than conventional antibiotics, repeated low-level exposure could still select for redox-tolerant microbial populations. Robust environmental fate studies, metagenomic surveillance, and longitudinal field data are largely lacking, making it premature to draw firm conclusions about ecosystem-level risk or benefit.

Taken together, these limitations do not negate the relevance of OSCs but rather delineate the conditions under which their development should proceed. Current evidence supports OSCs as mechanistically well-founded leads and adjunct candidates, not as near-term therapeutics. Addressing instability, PK variability, biomarker gaps, and ecological uncertainty through standardized, biomarker-driven, and context-specific approaches will be essential to bridge the gap between experimental promise and responsible pharmacological application.

## 12 Conclusion

Organosulfur compounds (OSCs) represent a promising class of dual-action therapeutic candidates, uniquely positioned to address antimicrobial resistance by simultaneously suppressing pathogenic fitness and enhancing host immune competence.

Their mechanistic versatility—combining thiol-reactive antimicrobial effects, metabolic disruption, membrane perturbation, efflux pump modulation, and quorum-sensing inhibition with host-directed activation of antioxidant (Nrf2) pathways and attenuation of pro-inflammatory signaling (NF-κB)—provides a rational basis for reduced microbial adaptability and improved infection outcomes. Despite compelling mechanistic evidence, the translational advancement of OSCs remains hindered by physicochemical instability, formulation limitations, and inconsistent systemic bioavailability. Inter-individual metabolic variability, off-target thiol reactivity, and incomplete toxicological characterization further limit clinical readiness. Addressing these issues requires targeted medicinal chemistry to enhance stability, validated pharmacokinetic/pharmacodynamic (PK/PD) markers, and biocompatible delivery platforms that preserve antimicrobial potency while minimizing host toxicity.

Moving forward, the most clinically prepared agents—such as stabilized isothiocyanates and engineered ajoene derivatives—should advance into well-controlled early-phase human studies, prioritized for localized applications (e.g., topical or mucosal administration) with clear PD endpoints such as biofilm load reduction, redox biomarker modulation, and cytokine signatures. Standardized in vitro–in vivo correlation models and proteomic profiling of thiol-modified targets will support rational dosing and safety assessment.

Finally, One Health implications underscore the importance of responsible OSC deployment to avoid unintended perturbations of animal microbiomes or environmental resistomes. Incorporating environmental fate studies and antimicrobial stewardship considerations early in development will ensure that OSC-based therapeutics evolve as sustainable, low-resistance-pressure interventions. Together, these aligned scientific and translational priorities define a realistic pathway for advancing OSCs from promising mechanistic leads toward evidence-based dual-action therapies. Future OSC development must balance mechanistic innovation with rigorous translational evaluation to ensure dual-action benefits translate sustainably across clinical and One Health contexts.

## References

[B1] Abd El-Ghany W. A. (2024). Potential effects of garlic (*Allium sativum* L.) on the performance, immunity, gut health, anti-oxidant status, blood parameters, and intestinal microbiota of poultry: an updated comprehensive review. Animals 14 (3), 498. 10.3390/ani14030498 38338142 PMC10854602

[B2] Acharya Y. Taneja K. K. Haldar J. (2023). Dual functional therapeutics: mitigating bacterial infection and associated inflammation. RSC Medicinal Chemistry 14 (8), 1410–1428. 10.1039/d3md00166k 37593575 PMC10429821

[B3] Albakr L. Du H. Zhang X. Kathuria H. Anwar‐Fadzil A. Wheate N. (2024). Progress in lipid and inorganic nanocarriers for enhanced skin drug delivery. Adv. NanoBiomed Res. 4, 1–24. 10.1002/anbr-202400003

[B4] Ali Redha A. Torquati L. Bows J. R. Gidley M. J. Cozzolino D. (2025). Microencapsulation of broccoli sulforaphane using whey and pea protein: *in vitro* dynamic gastrointestinal digestion and intestinal absorption by Caco-2-HT29-MTX-E12 cells. Food & Function 16 (1), 71–86. 10.1039/d4fo03446e 39431890

[B5] Alibrahem W. Nguyen D. H. H. Kharrat Helu N. Tóth F. Nagy P. T. Posta J. (2025). Health benefits, applications, and analytical methods of freshly produced Allyl isothiocyanate. Foods 14 (4), 579. 10.3390/foods14040579 40002023 PMC11853810

[B6] Alves I. Araújo E. M. Q. Dalgaard L. T. Singh S. Børsheim E. Carvalho E. (2025). Protective effects of sulforaphane preventing inflammation and oxidative stress to enhance metabolic health: a narrative review. Nutrients 17 (3), 428. 10.3390/nu17030428 39940284 PMC11821257

[B7] Alyasiri F. J. Ghobeh M. Tabrizi M. H. (2023). Preparation and characterization of allicin-loaded solid lipid nanoparticles surface-functionalized with folic acid-bonded chitosan: *in vitro* anticancer and antioxidant activities. Front. Bioscience (Landmark Edition) 28 (7), 135. 10.31083/j.fbl2807135 37525919

[B8] Andrés C. M. C. Pérez de la Lastra J. M. Andrés Juan C. Plou F. J. Pérez-Lebeña E. (2023). Chemistry of hydrogen sulfide-pathological and physiological functions in Mammalian cells. Cells 12 (23), 2684. 10.3390/cells12232684 38067112 PMC10705518

[B9] Andrés C. M. C. Pérez de la Lastra J. M. Bustamante Munguira E. Juan C. A. Plou F. J. Pérez Lebeña E. (2024). Electrophilic compounds in the human diet and their role in the induction of the transcription factor NRF2. Int. Journal Molecular Sciences 25 (6), 3521. 10.3390/ijms25063521 38542492 PMC10971185

[B10] Andrés C. M. C. Lobo F. Lastra J. M. P. d. l. Munguira E. B. Juan C. A. Pérez Lebeña E. (2025). Reactive Sulfur species and protein persulfidation: an emerging Redox axis in human health and disease. Curr. Issues Mol. Biol. 47 (9), 765. 10.3390/cimb47090765 41020887 PMC12468119

[B11] Apjok G. Számel M. Christodoulou C. Seregi V. Vásárhelyi B. Stirling T. (2023). Characterization of antibiotic resistomes by reprogrammed bacteriophage-enabled functional metagenomics in clinical strains. Nat. Microbiol. 8, 410–423. 10.1038/s41564-023-01320-2 36759752 PMC9981461

[B12] Arellano Buendia A. S. Juárez Rojas J. G. García-Arroyo F. Aparicio Trejo O. E. Sánchez-Muñoz F. Argüello-García R. (2023). Antioxidant and anti-inflammatory effects of allicin in the kidney of an experimental model of metabolic syndrome. PeerJ 11, e16132. 10.7717/peerj.16132 37786577 PMC10541809

[B13] Babiuch K. Kuśnierz-Cabala B. Kęsek B. Okoń K. Darczuk D. Chomyszyn-Gajewska M. (2020). Evaluation of proinflammatory, NF-kappaB dependent cytokines: Il-1α, IL-6, IL-8, and TNF-α in tissue specimens and saliva of patients with oral squamous cell carcinoma and oral potentially malignant disorders. J. Clin. Med. 9 (3), 867. 10.3390/jcm9030867 32245251 PMC7141524

[B14] Baralić K. Živanović J. Marić Đ. Bozic D. Grahovac L. Antonijević Miljaković E. (2024). Sulforaphane-A compound with potential health benefits for disease prevention and treatment: insights from pharmacological and toxicological experimental studies. Antioxidants. 13 (2), 147. 10.3390/antiox13020147 38397745 PMC10886109

[B15] Barman H. Kabir M. E. Borah A. Afzal N. U. Loying R. Sharmah B. (2025). “Therapeutic uses of dietary organosulfur compounds in response to viral (SARS-CoV-2)/Bacterial infection, inflammation, cancer, oxidative stress, cardiovascular diseases, obesity, and diabetes,”Chem. 22. e00731. 10.1002/cbdv.202500731 40857586

[B16] Barrett T. J. Pattison D. I. Leonard S. E. Carroll K. S. Davies M. J. Hawkins C. L. (2012). Inactivation of thiol-dependent enzymes by hypothiocyanous acid: role of sulfenyl thiocyanate and sulfenic acid intermediates. Free Radical Biology & Medicine 52 (6), 1075–1085. 10.1016/j.freeradbiomed.2011.12.024 22248862 PMC3523338

[B17] Bedir A. S. Almasri R. S. Azar Y. O. Elnady R. E. Al Raish S. M. (2025). Exploring the therapeutic potential of *Allium cepa* and *Allium sativum* extracts: current strategies, emerging applications, and sustainability utilization. Biology 14 (8), 1088. 10.3390/biology14081088 40906415 PMC12383551

[B18] Beleva E. Diukendjieva A. Pajeva I. Tsakovska I. (2025). Identification of compounds with potential dual inhibitory activity against drug efflux pumps in resistant cancer cells and bacteria: protocol for a systematic review. JMIR Research Protocols 14, e66197. 10.2196/66197 40472353 PMC12179561

[B19] Bell L. Wagstaff C. (2017). Enhancement of glucosinolate and isothiocyanate profiles in Brassicaceae crops: addressing challenges in breeding for cultivation, storage, and consumer-related traits. J. Agricultural Food Chemistry 65 (43), 9379–9403. 10.1021/acs.jafc.7b03628 28968493

[B20] Bellucci M. C. Romani C. Sani M. Volonterio A. (2024). Dual antibiotic approach: synthesis and antibacterial activity of antibiotic-antimicrobial peptide conjugates. Antibiot. 13 (8), 783. 10.3390/antibiotics13080783 39200083 PMC11352213

[B21] Bhatwalkar S. Mondal R. Krishna S. Adam J. Govender P. Anupam R. (2021). Antibacterial properties of organosulfur compounds of garlic (Allium sativum). Front. Microbiol. 12, 613077. 10.3389/fmicb.2021.613077 34394014 PMC8362743

[B22] Bhuiyan A. I. Papajani V. T. Paci M. Melino S. (2015). Glutathione-garlic sulfur conjugates: slow hydrogen sulfide-releasing agents for therapeutic applications. Mol. 20 (1), 1731–1750. 10.3390/molecules20011731 25608858 PMC6272329

[B23] Bianchi M. Winterhalter M. Harbig T. A. Hörömpöli D. Ghai I. Nieselt K. (2024). Fosfomycin uptake in *Escherichia coli* is mediated by the outer-membrane porins OmpF, OmpC, and LamB. ACS Infectious Diseases 10 (1), 127–137. 10.1021/acsinfecdis.3c00367 38104323 PMC10789261

[B24] Borlinghaus J. Albrecht F. Gruhlke M. C. Nwachukwu I. D. Slusarenko A. J. (2014). Allicin: chemistry and biological properties. Mol. 19 (8), 12591–12618. 10.3390/molecules190812591 25153873 PMC6271412

[B25] Borlinghaus J. Albrecht F. Gruhlke M. C. Nwachukwu I. D. Slusarenko A. J. (2021). Allicin: chemistry and biological property. Molecules 26 (1), 150. 10.3390/molecules190812591 PMC6271412 25153873

[B26] Bouranis D. Chorianopoulou S. (2023). Foliar application of sulfur-containing Compounds—Pros and cons. Plants 12, 3794. 10.3390/plants12223794 38005690 PMC10674314

[B27] Boutin C. Clément C. Rivoal J. (2024). Post-Translational modifications to cysteine residues in plant proteins and their impact on the regulation of metabolism and signal transduction. Int. Journal Molecular Sciences 25 (18), 9845. 10.3390/ijms25189845 39337338 PMC11432348

[B29] Busia K. (2024). Herbal medicine dosage standardization. J. Herb. Med. 46, 100889. 10.1016/j.hermed.2024.100889

[B30] Byrd B. A. Zenick B. Rocha-Granados M. C. Englander H. E. Hare P. J. LaGree T. J. (2021). The AcrAB-TolC efflux pump impacts persistence and resistance development in stationary-phase Escherichia coli following Delafloxacin treatment. Antimicrob. Agents Chemotherapy 65 (8), e0028121. 10.1128/AAC.00281-21 34097492 PMC8284433

[B31] Çakilli H. Ateş Ö. Taşpinar K. Kızılaslan F. (2025). Impacts of elemental sulfur application on soil microbial activity in alkaline calcareous soil. Geomicrobiol. J. 42, 576–580. 10.1080/01490451.2025.2490032

[B32] Cascajosa-Lira A. Andreo-Martínez P. Prieto A. I. Baños A. Guillamón E. Jos A. (2022). *In vitro* toxicity studies of bioactive organosulfur compounds from *Allium* spp. with potential application in the agri-food industry: a review. Foods. 11 (17), 2620. 10.3390/foods11172620 36076806 PMC9455835

[B33] Chaachouay N. Zidane L. (2024). Plant-Derived natural products: a source for drug discovery and development. Drugs Drug Candidates 3 (1), 184–207. 10.3390/ddc3010011

[B34] Chadha J. Harjai K. Chhibber S. (2022). Repurposing phytochemicals as anti-virulent agents to attenuate quorum-sensing-regulated virulence factors and biofilm formation in Pseudomonas aeruginosa. Microb. Biotechnology 15 (6), 1695–1718. 10.1111/1751-7915.13981 34843159 PMC9151347

[B35] Chavda V. P. Patel A. B. Mistry K. J. Suthar S. F. Wu Z.-X. Chen Z.-S. (2022). Nano-Drug delivery systems entrapping natural bioactive compounds for cancer: recent progress and future challenges. Front. Oncol. 12, 867655. 10.3389/fonc.2022.867655 35425710 PMC9004605

[B37] Chigozie V. U. Saki M. Esimone C. O. (2025a). Molecular structural arrangement in quorum sensing and bacterial metabolic production. World J. Microbiol. Biotechnol. 41, 71. 10.1007/s11274-025-04280-3 39939401

[B38] Chigozie V. U. Ugochukwu C. G. Igboji K. O. Okoye F. B. (2025b). Application of artificial intelligence in bioprospecting for natural products for biopharmaceutical purposes. BMC Artif. Intell. 1, 4. 10.1186/s44398-025-00004-7

[B39] Chigozie V. U. Aniokete U. C. Ogbonna I. P. Iroha I. R. (2025c). Transforming antimicrobial resistance mitigation: the genomic revolution in one health and public health. Discov. Appl. Sci. 7, 1187. 10.1007/s42452-025-07053-7

[B40] Cho S. J. Ryu J. H. Surh Y. J. (2019). Ajoene, a major organosulfide found in crushed garlic, induces NAD(P)H: quinone oxidoreductase expression through nuclear factor E2-related Factor-2 activation in human breast epithelial cells. J. Cancer Prev. 24 (2), 112–122. 10.15430/JCP.2019.24.2.112 31360690 PMC6619855

[B41] Choi S. J. McClements D. J. (2020). Nanoemulsions as delivery systems for lipophilic nutraceuticals: strategies for improving their formulation, stability, functionality, and bioavailability. Food Sci. Biotechnol. 29, 149–168. 10.1007/s10068-019-00731-4 32064124 PMC6992823

[B42] Choo S. Chin V. K. Wong E. H. Madhavan P. Tay S. T. Yong P. V. C. (2020). Review: antimicrobial properties of allicin used alone or in combination with other medications. Folia Microbiologica 65 (3), 451–465. 10.1007/s12223-020-00786-5 32207097

[B44] Corbu V. Gheorghe I. Marinaş I. Geană E. Moza M. Csutak O. (2021). Demonstration of Allium sativum extract inhibitory effect on biodeteriogenic microbial strain growth, biofilm development, and enzymatic and organic acid production. Molecules 26, 7195. 10.3390/molecules26237195 34885775 PMC8659052

[B45] Cruz K. Takumi O. Bongulto K. Gandalera E. Kagia N. Watanabe K. (2024). Natural compound-induced downregulation of antimicrobial resistance and biofilm-linked genes in wastewater Aeromonas species. Front. Cell. Infect. Microbiol. 14. 10.3389/fcimb.2024.1456700 PMC11513397 39469451

[B46] Dagah O. M. A. Silaa B. B. Zhu M. Pan Q. Qi L. Liu X. (2024). Exploring immune redox modulation in bacterial infections: insights into thioredoxin-mediated interactions and implications for understanding host-pathogen dynamics. Antioxidants. 13 (5), 545. 10.3390/antiox13050545 38790650 PMC11117976

[B48] Daley S. K. Cordell G. A. (2021). Alkaloids in contemporary drug Discovery to meet global disease needs. Mol. 26 (13), 3800. 10.3390/molecules26133800 34206470 PMC8270272

[B49] Danish Rizvi S. M. Abu Lila A. S. Moin A. Khafagy E. S. Rajab A. A. H. Hegazy W. A. H. (2025). Sulforaphane is not only a food supplement: it diminishes the intracellular survival and colonization of *Salmonella enterica* . ACS Omega 10 (3), 2969–2977. 10.1021/acsomega.4c09408 39895767 PMC11780411

[B50] De Groot A. Blanchard L. Rouhier N. Rey P. (2022). Thiol reductases in *Deinococcus* Bacteria and roles in stress tolerance. Antioxidants 11 (3), 561. 10.3390/antiox11030561 35326211 PMC8945050

[B240] Delgado D. L. C. Caceres L. L. C. Gómez S. A. C. Odio A. D. (2023). Effect of dietary garlic (Allium sativum) on the zootechnical performance and health indicators of aquatic animals: a mini-review. Veterinary world 16 (5), 965–976. 10.14202/vetworld.2023.965-976 37576751 PMC10420702

[B51] De Rossi L. Rocchetti G. Lucini L. Rebecchi A. (2025). Antimicrobial potential of polyphenols: mechanisms of action and microbial Responses—A narrative review. Antioxidants 14 (2), 200. 10.3390/antiox14020200 40002386 PMC11851925

[B52] Deng Y. Ho C. T. Lan Y. Xiao J. Lu M. (2023). Bioavailability, health benefits, and delivery systems of allicin: a review. J. Agricultural Food Chemistry 71 (49), 19207–19220. 10.1021/acs.jafc.3c05602 37943254

[B53] Dewanjee S. Bhattacharya H. Bhattacharyya C. Chakraborty P. Fleishman J. Alexiou A. (2024). Nrf2/Keap1/ARE regulation by plant secondary metabolites: a new horizon in brain tumor management. Cell Communication Signaling CCS 22 (1), 497. 10.1186/s12964-024-01878-2 39407193 PMC11476647

[B54] Ding Q. Ding L. Xiang C. Li C. Kim E. Yoon C. (2025a). pH-Responsive AIE photosensitizers for enhanced antibacterial therapy. Angewandte Chemie Int. Ed. Engl. 64 (27), e202506505. 10.1002/anie.202506505 40299633 PMC12207372

[B55] Ding Q. Qi M. Li W. Li M. Xu J. Kim Y. (2025b). Precision phototherapy enabled by decoding complex microenvironments. Accounts Chemical Research 58 (20), 3167–3183. 10.1021/acs.accounts.5c00488 41036779

[B56] Ding Q. Xie Y. Xiong K. Jia H. Chen L. Li C. (2025c). From light to cure: precision phototherapies for antibiotic-refractory biofilm infections. Angewandte Chemie Int. Ed. Engl. 64 (43), e202510900. 10.1002/anie.202510900 40944378

[B57] Dinkova-Kostova A. T. Copple I. M. (2023). Advances and challenges in therapeutic targeting of NRF2. Trends Pharmacological Sciences 44 (3), 137–149. 10.1016/j.tips.2022.12.003 36628798

[B58] Djamen C. Nyembo C. Ruben N. Tchouan G. Donfack M. Kana C. (2024). Effects of Allium sativum on growth performance, kidney and liver function markers, microbial flora and feed digestibility in broiler chickens. J. World’s Poult. Sci. 3, 1–11. 10.58803/jwps.v3i1.25

[B59] Dong X. Yu X. Lu M. Xu Y. Zhou L. Peng T. (2024). Quantitative chemical proteomics reveals that phenethyl isothiocyanate covalently targets BID to promote apoptosis. Cell Death Discovery 10 (1), 456. 10.1038/s41420-024-02225-7 39472556 PMC11522290

[B60] Duda-Madej A. Viscardi S. Pacyga K. Kupczyński R. Mączka W. Grabarczyk M. (2024). Antibiofilm and antimicrobial potentials of novel synthesized sulfur camphor derivatives. Int. J. Mol. Sci. 25 (20), 10895. 10.3390/ijms252010895 39456678 PMC11507198

[B61] Egbujor M. Petrosino M. Zuhra K. Saso L. (2022). The role of organosulfur compounds as Nrf2 activators and their antioxidant effects. Antioxidants 11, 1255. 10.3390/antiox11071255 35883746 PMC9311638

[B62] El-Saadony M. T. Saad A. M. Korma S. A. Salem H. M. Abd El-Mageed T. A. Alkafaas S. S. (2024). Garlic bioactive substances and their therapeutic applications for improving human health: a comprehensive review. Front. Immunology 15, 1277074. 10.3389/fimmu.2024.1277074 38915405 PMC11194342

[B63] ElKhalifa D. Al-Ziftawi N. Awaisu A. Alali F. Khalil A. (2023). Efficacy and tolerability of sulforaphane in the therapeutic management of cancers: a systematic review of randomized controlled trials. Front. Oncol. 13, 1251895. 10.3389/fonc.2023.1251895 38074675 PMC10710291

[B64] Fagiani F. Catanzaro M. Buoso E. Basagni F. Di Marino D. Raniolo S. (2020). Targeting cytokine release through the differential modulation of Nrf2 and NF-κB pathways by Electrophilic/Non-Electrophilic compounds. Front. Pharmacology 11, 1256. 10.3389/fphar.2020.01256 32922294 PMC7456937

[B65] Fahey J. W. Holtzclaw W. D. Wehage S. L. Wade K. L. Stephenson K. K. Talalay P. (2015). Sulforaphane bioavailability from Glucoraphanin-Rich broccoli: control by active endogenous myrosinase. PloS One 10 (11), e0140963. 10.1371/journal.pone.0140963 26524341 PMC4629881

[B66] Fahey J. W. Liu H. Batt H. Panjwani A. A. Tsuji P. (2025). Sulforaphane and brain health: from pathways of action to effects on specific disorders. Nutrients 17 (8), 1353. 10.3390/nu17081353 40284217 PMC12030691

[B67] Federici L. Masulli M. De Laurenzi V. Allocati N. (2025). A narrative review of the role of S-Glutathionylation in bacteria. Microorganisms 13 (3), 527. 10.3390/microorganisms13030527 40142423 PMC11944925

[B69] Firmino J. P. Galindo-Villegas J. Reyes-López F. E. Gisbert E. (2021). Phytogenic bioactive compounds shape fish mucosal immunity. Front. Immunology 12, 695973. 10.3389/fimmu.2021.695973 34220858 PMC8252966

[B70] Fitzsimons S. Muñoz-San Martín M. Nally F. Dillon E. Fashina I. A. Strowitzki M. J. (2023). Inhibition of pro-inflammatory signaling in human primary macrophages by enhancing arginase-2 *via* target site blockers. Mol. Therapy. Nucleic Acids 33, 941–959. 10.1016/j.omtn.2023.08.023 37701067 PMC10494319

[B72] Gao W. Guo L. Yang Y. Wang Y. Xia S. Gong H. (2022). Dissecting the crosstalk between Nrf2 and NF-κB response pathways in drug-induced toxicity. Front. Cell Developmental Biology 9, 809952. 10.3389/fcell.2021.809952 35186957 PMC8847224

[B74] Gbala I. D. Macharia R. W. Bargul J. L. Magoma G. (2022). Membrane permeabilization and antimicrobial activity of recombinant Defensin-d2 and actifensin against multidrug-resistant *Pseudomonas aeruginosa* and *Candida albicans* . Mol. 27 (14), 4325. 10.3390/molecules27144325 35889198 PMC9317813

[B75] Gerson J. Hinckley E. (2023). It is time to develop sustainable management of agricultural sulfur. Earth's Future 11, e2023EF003723. 10.1029/2023ef003723

[B78] Giron J. Smiarowski L. Katz J. (2024). The effect of sulforaphane on markers of inflammation and metabolism in virally suppressed HIV patients. Front. Nutr. 11, 1357906. 10.3389/fnut.2024.1357906 39539366 PMC11557404

[B79] Gong Z. Yu H. Zhang J. Li F. Song H. (2020). Microbial electro-fermentation for the synthesis of chemicals and biofuels driven by bi-directional extracellular electron transfer. Synthetic Syst. Biotechnol. 5, 304–313. 10.1016/j.synbio.2020.08.004 32995586 PMC7490822

[B80] Gong Q. Wang X. Liu Y. Yuan H. Ge Z. Li Y. (2024). Potential hepatoprotective effects of Allicin on carbon tetrachloride-induced acute liver injury in mice by inhibiting oxidative stress, inflammation, and apoptosis. Toxics 12 (5), 328. 10.3390/toxics12050328 38787107 PMC11126064

[B81] González F. Quintero J. Del Río R. Mahn A. (2021). Optimization of an extraction process to obtain a food-grade sulforaphane-rich extract from broccoli (Brassica oleracea var. italica). Molecules 26, 4042. 10.3390/molecules26134042 34279379 PMC8272218

[B82] Grady R. S. Traustadóttir T. Lagalante A. F. Eggler A. L. (2023). Bioavailable sulforaphane quantitation in plasma by LC-MS/MS is enhanced by blocking thiols. J. Agricultural Food Chemistry 71 (34), 12875–12882. 10.1021/acs.jafc.3c01367 37584212 PMC10472501

[B84] Gruhlke M. C. H. Antelmann H. Bernhardt J. Kloubert V. Rink L. Slusarenko A. J. (2019). The human allicin-proteome: S-thioallylation of proteins by the garlic defence substance allicin and its biological effects. Free Radical Biology & Medicine 131, 144–153. 10.1016/j.freeradbiomed.2018.11.022 30500420 PMC6342545

[B85] Guillamón E. Andreo-Martínez P. Mut-Salud N. Fonollá J. Baños A. (2021). Beneficial effects of organosulfur compounds from *Allium cepa* on gut health: a systematic review. Foods 10 (8), 1680. 10.3390/foods10081680 34441457 PMC8392556

[B86] Guo Y. Li Z. Xu P. Guo G. He T. Lai Y. (2025). Subchronic and chronic toxicity assessment of Sublancin in sprague-dawley rats. Toxics 13 (5), 413. 10.3390/toxics13050413 40423492 PMC12115613

[B87] Habtemariam S. (2024). Anti-Inflammatory therapeutic mechanisms of isothiocyanates: insights from sulforaphane. Biomedicines 12 (6), 1169. 10.3390/biomedicines12061169 38927376 PMC11200786

[B88] Hadjicharalambous A. Bournakas N. Newman H. Skynner M. J. Beswick P. (2022). Antimicrobial and cell-penetrating peptides: understanding penetration for the design of novel conjugate antibiotics. Antibiot. 11 (11), 1636. 10.3390/antibiotics11111636 36421280 PMC9686638

[B89] Hanek K. Żak P. (2024). Eco-Friendly functionalization of ynals with thiols under mild conditions. Int. J. Mol. Sci. 25, 9201. 10.3390/ijms25179201 39273150 PMC11395323

[B90] Hermes A. Logan M. Poulin B. McKenna A. Dawson T. Borch T. (2023). Agricultural Sulfur applications Alter the quantity and composition of dissolved organic matter from field-to-watershed scales. Environ. Science & Technology 57, 10019–10029. 10.1021/acs.est.3c01347 37382932

[B91] Herzog I. M. Green K. D. Berkov-Zrihen Y. Feldman M. Vidavski R. R. Eldar-Boock A. (2012). 6''-Thioether tobramycin analogues: towards selective targeting of bacterial membranes. Angewandte Chemie Int. Ed. Engl. 51 (23), 5652–5656. 10.1002/anie.201200761 22499286 PMC3390782

[B92] Hickson S. M. Ledger E. L. Wells T. J. (2025). Emerging antimicrobial therapies for Gram-negative infections in human clinical use. Npj Antimicrobials Resistance 3 (1), 16. 10.1038/s44259-025-00087-2 40016340 PMC11868545

[B93] Hu W. Huang L. Zhou Z. Yin L. Tang J. (2022). Diallyl disulfide (DADS) ameliorates intestinal *Candida albicans* infection by modulating the gut microbiota and metabolites and providing intestinal protection in mice. Front. Cell. Infect. Microbiol. 11, 743454. 10.3389/fcimb.2021.743454 35071031 PMC8777027

[B94] Hu K. Wang Z. Xu Q. Chu Y. Qian Y. Zhang X. (2025). Metagenomic insights into the effects of ecological water replenishment on resistome and pathogens in urban wetland. J. Hazardous Materials 499, 140117. 10.1016/j.jhazmat.2025.140117 41129998

[B174] Igwe O. F. Briggs T. A. Dike U. A. Okata-Nwachukwu M. O. Akpogene C. M. Ugboaja F. C. (2025). A pilot study of the antibacterial activity of garlic (Allium Sativum) against selected drug resistant organisms. IJRSI 12 (15), 618–637. 10.51244/IJRSI.2025.121500056P

[B95] Islam F. Zeng Q. (2024). Advances in organosulfur-based polymers for drug delivery systems. Polymers 16 (9), 1207. 10.3390/polym16091207 38732676 PMC11085353

[B96] Ismarica I. Helda M. Dewi D. (2025). Addition of garlic (Allium sativum) extract in fry as an immunostimulant on survival and blood profile of pomfret fish (Colossoma macropomum). BIO Web Conf. 156. 10.1051/bioconf/202515603027

[B97] Jakobsen T. H. van Gennip M. Phipps R. K. Shanmugham M. S. Christensen L. D. Alhede M. (2012). Ajoene, a sulfur-rich molecule from garlic, inhibits genes controlled by quorum sensing. Antimicrob. Agents Chemotherapy 56 (5), 2314–2325. 10.1128/AAC.05919-11 22314537 PMC3346669

[B98] Janczewski Ł. (2022). Sulforaphane and its bifunctional analogs: synthesis and biological activity. Mol. 27 (5), 1750. 10.3390/molecules27051750 35268851 PMC8911885

[B99] Jara G. Terol G. Martínez S. Cánovas M. de Diego T. (2021). Engineering of microbial cell factories for the production of plant-based natural products. 10.1016/B978-0-12-821477-0.00019-2

[B101] Jikah A. N. Edo G. I. Makia R. Yousif E. Gaaz T. S. Isoje E. F. (2024). A review of the therapeutic potential of sulfur compounds in Allium sativum. Meas. Food. 15, 100195. 10.1016/j.meafoo.2024.100195

[B104] Keenum I. Wind L. Ray P. Guron G. Chen C. Knowlton K. (2022). Metagenomic tracking of antibiotic resistance genes through a pre‐harvest vegetable production system: an integrated lab‐, microcosm‐ and greenhouse‐scale analysis. Environ. Microbiol. 24, 3705–3721. 10.1111/1462-2920.16022 35466491 PMC9541739

[B105] Kim K. S. Han C. Y. Han Y. T. Bae E. J. (2019). Rhodanthpyrone A and B play an anti-inflammatory role by suppressing the nuclear factor-κB pathway in macrophages. Korean Soc. Pharmacol. 23 (6), 493–499. 10.4196/kjpp.2019.23.6.493 31680771 PMC6819899

[B106] Kim J. H. Cheng L. W. Land K. M. Gruhlke M. C. H. (2021). Editorial: Redox-Active molecules as antimicrobials: mechanisms and resistance. Front. Microbiology 12, 758750. 10.3389/fmicb.2021.758750 34566946 PMC8461237

[B107] Kim H. R. Ingram J. L. Que L. G. (2023). Effects of oxidative stress on airway epithelium permeability in asthma and potential implications for patients with comorbid obesity. J. Asthma Allergy 16, 481–499. 10.2147/JAA.S402340 37181453 PMC10171222

[B108] Kim J. Y. Jee H. G. Kim J. Y. Yong T. S. Jeon S. H. (2024). NF-κB p65 and TCF-4 interactions are associated with LPS-stimulated IL-6 secretion of macrophages. Biochem. Biophysics Reports 38, 101659. 10.1016/j.bbrep.2024.101659 38352245 PMC10859262

[B109] Kim J. K. Sapkota A. Roh T. Jo E. K. (2025). The intricate interactions between inflammasomes and bacterial pathogens: roles, mechanisms, and therapeutic potentials. Pharmacol. Ther. 265, 108756. 10.1016/j.pharmthera.2024.108756 39581503

[B110] Kim S. Y. Kim J. M. Chung K. S. Jang D. S. Lee J. Y. Kim C. (2025). *In vitro* and *in vivo* anti-inflammatory effects of 5-hydroxyconiferaldehyde *via* NF-κB, MAPK/AP-1, and Nrf2 modulation. Chem.-Biol. Interact. 409, 111427. 10.1016/j.cbi.2025.111427 39956256

[B111] Ko H. S. Kim K. Na Y. R. Yeom C. H. Nho C. W. Cho Y. S. (2025). Phenethyl Isothiocyanate (PEITC) interaction with Keap1 activates the Nrf2 pathway and inhibits lipid accumulation in adipocytes. J. Nutr. Biochem. 144, 109963. 10.1016/j.jnutbio.2025.109963 40383280

[B112] Kolygas M. N. Bitchava K. Nathanailides C. Athanassopoulou F. (2025). Phytochemicals: essential oils and other extracts for disease prevention and growth enhancement in aquaculture: challenges and opportunities. Animals 15 (18), 2653. 10.3390/ani15182653 41007897 PMC12466357

[B113] Kosuge Y. (2020). Neuroprotective mechanisms of S-allyl-L-cysteine in neurological disease. Exp. Ther. Med. 19, 1565–1569. 10.3892/etm.2019.8391 32010340 PMC6966174

[B114] Kothari D. Lee W.-D. Niu K.-M. Kim S.-K. (2019). The genus allium as poultry feed additive: a review. Animals 9 (12), 1032. 10.3390/ani9121032 31779230 PMC6940947

[B115] Kumadoh D. N'guessan B. Adase E. Sarkodie J. (2023). Batch-to-batch consistency in the quality attributes of a phyto-pharmaceutical MA001 used to treat typhoid in Ghana. J. Med. Biomed. Sci. 9, 1–17. 10.54106/249509.jmbs7z

[B118] Lai Y. H. Chiang Y. F. Huang K. C. Chen H. Y. Ali M. Hsia S. M. (2023). Allyl isothiocyanate mitigates airway inflammation and constriction in a house dust mite-induced allergic asthma model *via* upregulation of tight junction proteins and the TRPA1 modulation. Biomed. Pharmacother. 166, 115334. 10.1016/j.biopha.2023.115334 37634475

[B119] Langston-Cox A. Anderson D. Creek D. J. Palmer K. Wallace E. M. Marshall S. A. (2020). Measuring sulforaphane and its metabolites in human plasma: a high throughput method. Molecules 25 (4), 829. 10.3390/molecules25040829 32070059 PMC7070302

[B120] Lawson L. D. Hunsaker S. M. (2018). Allicin bioavailability and bioequivalence from garlic supplements and garlic foods. Nutrients 10 (7), 812. 10.3390/nu10070812 29937536 PMC6073756

[B121] Li W. Zeng T. Yao J. Zhu L. Zhang Z. Xie X. (2020). Diallyl sulfide from garlic suppresses quorum‐sensing systems of Pseudomonas aeruginosa and enhances biosynthesis of three B vitamins through its thioether group. Microb. Biotechnol. 14, 677–691. 10.1111/1751-7915.13829 33377615 PMC7936293

[B122] Li X. Ni M. Xu X. Chen W. (2020). Characterisation of naturally occurring isothiocyanates as glutathione reductase inhibitors. J. Enzyme Inhibition Medicinal Chemistry 35 (1), 1773–1780. 10.1080/14756366.2020.1822828 32951477 PMC7534374

[B123] Li W. R. Zeng T. H. Yao J. W. Zhu L. P. Zhang Z. Q. Xie X. B. (2021). Diallyl sulfide from garlic suppresses quorum-sensing systems of Pseudomonas aeruginosa and enhances biosynthesis of three B vitamins through its thioether group. Microb. Biotechnology 14 (2), 677–691. 10.1111/1751-7915.13729 33377615 PMC7936293

[B125] Li X. Wang Y. Zhao G. Liu G. Wang P. Li J. (2022). Microorganisms—An effective tool to intensify the utilization of sulforaphane. Foods 11 (23), 3775. 10.3390/foods11233775 36496582 PMC9737538

[B127] Li S. Wang Y. Xu G. Xu Y. Fu C. Zhao Q. (2024). The combination of allicin with domiphen is effective against microbial biofilm formation. Front. Microbiol. 15, 1341316. 10.3389/fmicb.2024.1341316 38873153 PMC11169630

[B128] Li M. Zhan A. Rahman T. Jiang T. Hou L. (2025). From wastewater to resistance: characterization of multidrug-resistant bacteria and assessment of natural antimicrobial compounds. Front. Microbiol. 16, 1612534. 10.3389/fmicb.2025.1612534 40708915 PMC12286950

[B129] Lian S. Liu J. Yang Y. Zhu G. Xia P. (2025). The life-and-death struggle between the complement system and pathogens: mechanisms of elimination, evasion tactics, and translational potential. Virulence 16 (1), 2553781. 10.1080/21505594.2025.2553781 40905313 PMC12413066

[B130] Liang X. Zhang Y. Wan S. Xia L. Li P. Wang S. (2025). Impacts of sulfur application on microbial communities and functional attributes in rubber plantation soil. BMC Microbiology 25 (1), 265. 10.1186/s12866-025-03971-z 40316901 PMC12046827

[B131] Lii C. K. Liu K. L. Cheng Y. P. Lin A. H. Chen H. W. Tsai C. W. (2010). Sulforaphane and alpha-lipoic acid upregulate the expression of the pi class of glutathione S-transferase through c-Jun and Nrf2 activation. J. Nutr. 140 (5), 885–892. 10.3945/jn.110.121418 20237067

[B132] Liu Z. Lei J. Zhang X. Yin J. Zhang Y. Lei K. (2025). Evolution of ceftazidime-avibactam resistance driven by variation in *bla* _KPC-2_ to *bla* _KPC-190_ during treatment of ST11-K64 hypervirulent *Klebsiella pneumoniae* . Front. Cell. Infect. Microbiol. 15, 1607127. 10.3389/fcimb.2025.1607127 40546282 PMC12179193

[B133] Loh W. Vermeren S. (2022). Anti-Inflammatory neutrophil functions in the resolution of inflammation and tissue repair. Cells 11 (24), 4076. 10.3390/cells11244076 36552840 PMC9776979

[B134] Loi V. V. Rossius M. Antelmann H. (2015). Redox regulation by reversible protein S-thiolation in bacteria. Front. Microbiology 6, 187. 10.3389/fmicb.2015.00187 25852656 PMC4360819

[B135] Loi V. V. Huyen N. T. T. Busche T. Tung Q. N. Gruhlke M. C. H. Kalinowski J. (2019). Staphylococcus aureus responds to allicin by global S-thioallylation - role of the Brx/BSH/YpdA pathway and the disulfide reductase MerA to overcome allicin stress. Free Radic. Biol. Med. 139, 55–69. 10.1016/j.freeradbiomed.2019.05.018 31121222

[B136] Lu W. (2024). Sulforaphane regulates AngII-induced podocyte oxidative stress injury through the Nrf2-Keap1/ho-1/ROS pathway. Ren. Failure 46 (2), 2416937. 10.1080/0886022X.2024.2416937 39417305 PMC11488169

[B137] Lu X. Samuelson D. R. Rasco B. A. Konkel M. E. (2012). Antimicrobial effect of diallyl sulphide on Campylobacter jejuni biofilms. J. Antimicrob. Chemother. 67 (8), 1915–1926. 10.1093/jac/dks138 22550133 PMC3394439

[B138] Luo Q. Ding N. Liu Y. Zhang H. Fang Y. Yin L. (2023). Metabolic engineering of microorganisms to produce pyruvate and derived compounds. Molecules 28, 1418. 10.3390/molecules28031418 36771084 PMC9919917

[B139] Ma C. Gu C. Lian P. Wazir J. Lu R. Ruan B. (2023). Sulforaphane alleviates psoriasis by enhancing antioxidant defense through the KEAP1-NRF2 Pathway activation and attenuating inflammatory signaling. Cell Death Dis. 14 (11), 768. 10.1038/s41419-023-06234-9 38007430 PMC10676357

[B140] Macesic N. Uhlemann A. C. Peleg A. Y. (2025). Multidrug-resistant Gram-negative bacterial infections. Lancet. 405 (10474), 257–272. 10.1016/S0140-6736(24)02081-6 39826970

[B141] Makuch A. Ziomek M. Sapała M. Drabik K. Batkowska J. Domaradzki P. (2025). The impact of allicin on the growth of *clostridium* spp. in the digestive track of quails. Animals 15 (7), 906. 10.3390/ani15070906 40218300 PMC11988147

[B142] Marefati N. Ghorani V. Shakeri F. Boskabady M. Kianian F. Rezaee R. (2021). A review of anti-inflammatory, antioxidant, and immunomodulatory effects of *Allium cepa* and its main constituents. Pharm. Biology 59 (1), 287–302. 10.1080/13880209.2021.1874028 33645419 PMC7919894

[B143] Martins N. Petropoulos S. Ferreira I. C. (2016). Chemical composition and bioactive compounds of garlic (Allium sativum L.) as affected by pre- and post-harvest conditions: a review. Food Che. 211, 41–50. 10.1016/j.foodchem.2016.05.029 27283605

[B144] Mayer C. Riera-Ponsati L. Kauppinen S. Klitgaard H. Erler J. T. Hansen S. N. (2024). Targeting the NRF2 pathway for disease modification in neurodegenerative diseases: mechanisms and therapeutic implications. Front. Pharmaco. 15, 1437939. 10.3389/fphar.2024.1437939 39119604 PMC11306042

[B145] Meng Q. T. Chen R. Chen C. Su K. Li W. Tang L. H. (2017). Transcription factors Nrf2 and NF-κB contribute to inflammation and apoptosis induced by intestinal ischemia-reperfusion in mice. Int. J. Molecu. Medi. 40 (6), 1731–1740. 10.3892/ijmm.2017.3170 29039475 PMC5716448

[B146] Michaelis C. Grohmann E. (2023). Horizontal gene transfer of antibiotic resistance genes in biofilms. Antibiotics 12, 328. 10.3390/antibiotics12020328 36830238 PMC9952180

[B147] Mitra S. Das R. Emran T. B. Labib R. K. Islam F. Sharma R. (2022). Diallyl disulfide: a bioactive garlic compound with anticancer potential. Front. Pharmacol. 13, 943967. 10.3389/fphar.2022.943967 36071845 PMC9441672

[B148] Mösbauer K. Fritsch V. N. Adrian L. Bernhardt J. Gruhlke M. C. H. Slusarenko A. J. (2021). The effect of allicin on the proteome of SARS-CoV-2 infected Calu-3 cells. Front. Microbiol. 12, 746795. 10.3389/fmicb.2021.746795 34777295 PMC8581659

[B149] Müller A. Eller J. Albrecht F. Prochnow P. Kuhlmann K. Bandow J. E. (2016). Allicin induces Thiol stress in bacteria through S-Allylmercapto modification of protein cysteines. J. Biol. Chem. 291 (22), 11477–11490. 10.1074/jbc.M115.702308 27008862 PMC4882420

[B150] Munusamy S. Conde R. Bertrand B. Munoz-Garay C. (2020). Biophysical approaches for exploring lipopeptide-lipid interactions. Biochimie 170, 173–202. 10.1016/j.biochi.2020.01.009 31978418 PMC7116911

[B151] Murugaiyan J. Kumar P. A. Rao G. S. Iskandar K. Hawser S. Hays J. P. (2022). Progress in alternative strategies to combat antimicrobial resistance: focus on antibiotics. Antibiot. 11 (2), 200. 10.3390/antibiotics11020200 35203804 PMC8868457

[B152] Nakamoto M. Kunimura K. Suzuki J. I. Kodera Y. (2020). Antimicrobial properties of hydrophobic compounds in garlic: allicin, vinyldithiin, ajoene, and diallyl polysulfides. Exp. Therapeutic Medicine 19 (2), 1550–1553. 10.3892/etm.2019.8388 32010337 PMC6966194

[B153] Nakamoto M. Kunimura K. Ohtani M. (2025). Pharmacokinetics of sulfur? Containing compounds in aged garlic extract: *S*? Allylcysteine, *S*?1?propenylcysteine, *S*? Methylcysteine, *S*? Allylmercaptocysteine, and others. Exp. Therapeutic Medicine 29 (5), 102. 10.3892/etm.2025.12852 40171136 PMC11959343

[B154] Napit R. Gurung A. Poudel A. Chaudhary A. Manandhar P. Sharma A. (2025). Metagenomic analysis of human, animal, and environmental samples identifies potential emerging pathogens, profiles antibiotic resistance genes, and reveals horizontal gene transfer dynamics. Sci. Rep. 15, 12156. 10.1038/s41598-025-90777-8 40204742 PMC11982193

[B155] Nikou T. Sakavitsi M. E. Kalampokis E. Halabalaki M. (2022). Metabolism and bioavailability of olive bioactive constituents based on *in vitro,*, *in vivo,*, and human studies. Nutrients 14 (18), 3773. 10.3390/nu14183773 36145149 PMC9504511

[B156] Noor T. Schultz D. C. Seabra G. Zhai Y. Jeong K. C. Bokhari S. A. (2025). Synthesis, structural studies, and inhibitory potential of selected sulfonamide analogues: insights from *in silico* and *in vitro* analyses. EXCLI Journal 24, 527–538. 10.17179/excli2024-8118 40376437 PMC12078777

[B157] Olayanju J. B. Bozic D. Naidoo U. Sadik O. A. (2024). A comparative review of key isothiocyanates and their health benefits. Nutrients 16 (6), 757. 10.3390/nu16060757 38542669 PMC10974736

[B158] Oluwabusola E. T. Katermeran N. P. Poh W. H. Goh T. M. B. Tan L. T. Diyaolu O. (2022). Inhibition of the quorum sensing System, elastase production, and biofilm Formation in *Pseudomonas aeruginosa* by Psammaplin A and bisaprasin. Molecules 27 (5), 1721. 10.3390/molecules27051721 35268822 PMC8911947

[B159] Onisei T. Tihăuan B. M. Dolete G. Axinie Bucos M. Răscol M. Isvoranu G. (2023). *In vivo* acute toxicity and immunomodulation assessment of a novel nutraceutical in mice. Pharmaceutics 15 (4), 1292. 10.3390/pharmaceutics15041292 37111777 PMC10144505

[B160] Ottosen C. Bjerg P. Kümmel S. Richnow H. Middeldorp P. Draborg H. (2024). Natural attenuation of sulfonamides and metabolites in contaminated groundwater - review, advantages and challenges of current documentation techniques. Water Research 254, 121416. 10.1016/j.watres.2024.121416 38489851

[B161] Ouyang Q. Zhou H. Yu Z. Jiang H. Ji C. Sun Y. (2025). IKK/NF-κB inactivation by salidroside *via* targeting TNF-α for the treatment of LPS-Induced colitis. Curr. Issues Mol. Biol. 47 (11), 896. 10.3390/cimb47110896 41296400 PMC12651885

[B162] Pan Y. Matsunaga T. Zhang T. Akaike T. (2025). The therapeutic potential of supersulfides in oxidative stress-related diseases. Biomolecules 15 (2), 172. 10.3390/biom15020172 40001475 PMC11852411

[B163] Park W. S. Lee J. Na G. Park S. Seo S.-K. Choi J. S. (2022). Benzyl isothiocyanate attenuates inflammasome activation in *Pseudomonas aeruginosa* LPS-Stimulated THP-1 cells and exerts regulation through the MAPKs/NF-κB pathway. Int. J. Mol. Sci. 23 (3), 1228. 10.3390/ijms23031228 35163151 PMC8835927

[B164] Patel V. Dial K. Wu J. Gauthier A. G. Wu W. Lin M. (2020). Dietary antioxidants significantly attenuate Hyperoxia-Induced acute inflammatory lung injury by enhancing macrophage function *via* reducing the accumulation of airway HMGB1. Int. Journal Molecular Sciences 21 (3), 977. 10.3390/ijms21030977 32024151 PMC7037000

[B165] Patel B. Greenland J. C. Williams-Gray C. H. (2024). Clinical trial highlights: Anti-Inflammatory and immunomodulatory agents. J. Parkinson's Disease 14 (7), 1283–1300. 10.3233/JPD-240353 39331111 PMC11492043

[B166] Patel P. Garala K. Singh S. Prajapati B. G. Chittasupho C. (2024). Lipid-Based nanoparticles in delivering bioactive compounds for improving therapeutic efficacy. Pharmaceuticals 17 (3), 329. 10.3390/ph17030329 38543115 PMC10975431

[B168] Percio A. Cicchinelli M. Masci D. Summo M. Urbani A. Greco V. (2024). Oxidative cysteine post-translational modifications drive the Redox code underlying neurodegeneration and amyotrophic lateral sclerosis. Antioxidants 13 (8), 883. 10.3390/antiox13080883 39199129 PMC11351139

[B169] Pötschke V. Bereswill S. Heimesaat M. M. (2025). Antibacterial effects of sulforaphane - a phytonutrient derived from broccoli as a promising candidate in the combat of bacterial infections. Eur. J. Microbiol. Immunol. 15 (3), 139–149. 10.1556/1886.2025.00028 40553560 PMC12505145

[B170] Pouremamali F. Pouremamali A. Dadashpour M. Soozangar N. Jeddi F. (2022). An update of Nrf2 activators and inhibitors in cancer prevention/promotion. Cell. Commun. Signal. 20 (1), 100. 10.1186/s12964-022-00906-3 35773670 PMC9245222

[B171] Pun M. Khazanov N. Galsurker O. Kerem Z. Senderowitz H. Yedidia I. (2023). Inhibition of AcrAB-TolC enhances antimicrobial activity of phytochemicals in *Pectobacterium brasiliense* . Front. Plant Sci. 14, 1161702. 10.3389/fpls-2023.1161702 37229130 PMC10203483

[B172] Qadri H. Haseeb Shah A. Mudasir Ahmad S. Alshehri B. Almilaibary A. Ahmad Mir M. (2022). Natural products and their semi-synthetic derivatives against antimicrobial-resistant human pathogenic bacteria and fungi. Saudi J. Biol. Sci. 29 (9), 103376. 10.1016/j.sjbs.2022.103376 35874656 PMC9290337

[B173] Qiu X. Wang B. Ren S. Liu X. Wang Y. (2024). Regulation of quorum sensing for the manipulation of conjugative transfer of antibiotic resistance genes in the wastewater treatment system. Water Research 253, 121222. 10.1016/j.watres.2024.121222 38335841

[B374] Rahmatinia E. Amidi B. Naderi N. Ahmadipour S. Ahmadvand H. Fallahy P. (2024). Randomized, double-blind clinical trial evaluating the impact of freeze-dried garlic extract capsules on blood pressure, lipid profile, and nitric oxide levels in individuals at risk for hypertension. Horm. Mol. Biol. Clin. Investig. 45, 139–147. 10.1515/hmbci-2024-0019 39001718

[B175] Rahul Sandeep T. Sravya M. V. N. Simhachalam G. (2025). Phytobiotics in finfish and shellfish: a systematic review. Discov. Anim. 2, 74. 10.1007/s44338-025-00098-3

[B176] Reiter J. Levina N. Van der Linden M. Gruhlke M. Martin C. Slusarenko A. J. (2017). Diallylthiosulfinate (Allicin), a volatile antimicrobial from Garlic (*Allium sativum*), kills human lung pathogenic bacteria, including MDR strains, as a vapor. Molecules 22 (10), 1711. 10.3390/molecules22101711 29023413 PMC6151386

[B177] Robinson C. Carbonell P. Jervis A. Yan C. Hollywood K. Dunstan M. (2020). Rapid prototyping of microbial production strains for the biomanufacture of potential materials monomers. Metab. Eng. 60, 168–182. 10.1016/j.ymben.2020.04.008 32335188 PMC7225752

[B178] Romero-Durán M. A. Silva-García O. Pérez-Aguilar J. M. Baizabal-Aguirre V. M. (2024). Mechanisms of Keap1/Nrf2 modulation in bacterial infections: implications in persistence and clearance. Front. Immunology 15, 1508787. 10.3389/fimmu.2024.1508787 39763664 PMC11700987

[B179] Rouf R. Uddin S. J. Sarker D. K. Islam M. T. Ali E. S. Shilpi J. A. (2020). Antiviral potential of garlic (*Allium sativum*) and its organosulfur compounds: a systematic update of pre-clinical and clinical data. Trends Food Sci. Technol. 104, 219–234. 10.1016/j.tifs.2020.08.006 32836826 PMC7434784

[B181] Ruhee R. T. Roberts L. A. Ma S. Suzuki K. (2020). Organosulfur compounds: a review of their anti-inflammatory effects in human health. Front. Nutr. 7, 64. 10.3389/fnut.2020.00064 32582751 PMC7280442

[B182] Russo C. Mesini A. Mariani M. Tavella E. Sette C. Ugolotti E. (2024). Reduce susceptibility to cefiderocol in gram-negative bacteria in children: is hope already lost before it's even arrived? J. Infection Public Health 17 (4), 624–631. 10.1016/j.jiph.2024.02.006 38422857

[B183] Sadanov A. K. Baimakhanova B. B. Orasymbet S. E. Ratnikova I. A. Turlybaeva Z. Z. Baimakhanova G. B. (2025). Engineering useful microbial species for pharmaceutical applications. Microorganisms 13 (3), 599. 10.3390/microorganisms13030599 40142492 PMC11944651

[B184] Saito A. Ishikawa S. Yang K. Sawa A. Ishizuka K. (2025). Sulforaphane as a potential therapeutic agent: a comprehensive analysis of clinical trials and mechanistic insights. J. Nutritional Sci. 14, e65. 10.1017/jns.2025.10033 40988712 PMC12451241

[B185] Salam M. A. Al-Amin M. Y. Salam M. T. Pawar J. S. Akhter N. Rabaan A. A. (2023). Antimicrobial resistance: a growing serious threat for global public health. Healthc. 11 (13), 1946. 10.3390/healthcare11131946 37444780 PMC10340576

[B186] Salehi B. Zucca P. Orhan I. E. Azzini E. Adetunji C. O. Mohammed S. A. (2019). Allicin and health: a comprehensive review. Trends Food Sci. & Technol. 86, 502–516. 10.1016/j.tifs.2019.03.003

[B187] Sánchez C. J. Martínez-Miró S. Ariza J. J. Madrid J. Orengo J. Aguinaga M. A. (2020). Effect of *Alliaceae* extract supplementation on performance and intestinal microbiota of growing-finishing pig. Animals An Open Access Journal MDPI 10 (9), 1557. 10.3390/ani10091557 32887323 PMC7552321

[B188] Sarangi A. Das B. S. Pahuja I. Ojha S. Singh V. Giri S. (2024). Ajoene: a natural compound with enhanced antimycobacterial and antibiofilm properties mediated by efflux pump modulation and ROS generation against M. Smegmatis. Archives Microbiology 206 (12), 453. 10.1007/s00203-024-04189-9 39487375

[B189] Sarshar M. Behzadi P. Ambrosi C. Zagaglia C. Palamara A. T. Scribano D. (2020). FimH and anti-adhesive therapeutics: a disarming strategy against uropathogens. Antibiotics 9 (7), 397. 10.3390/antibiotics9070397 32664222 PMC7400442

[B190] Schepetkin I. A. Kirpotina L. N. Khlebnikov A. I. Balasubramanian N. Quinn M. T. (2019). Neutrophil immunomodulatory activity of natural organosulfur compounds. Mol. 24 (9), 1809. 10.3390/molecules24091809 31083328 PMC6539273

[B191] Schuergers N. Werlang C. Ajo-Franklin C. M. Boghossian A. A. (2017). A synthetic biology approach to engineering living photovoltaics. Energy Environ. Sci. 10 (5), 1102–1115. 10.1039/C7EE00282C 28694844 PMC5501249

[B192] Sead F. Jain V. Kumar A. M. R. Kundlas M. Gupta S. Kumari M. (2025). Magnetically recoverable catalysts for efficient multicomponent synthesis of organosulfur compounds. RSC Adv. 15, 3928–3953. 10.1039/d4ra08769k 39917045 PMC11799890

[B193] Shang A. Cao S. Y. Xu X. Y. Gan R. Y. Tang G. Y. Corke H. (2019). Bioactive compounds and biological functions of garlic (*Allium sativum* L.). Foods 8 (7), 246. 10.3390/foods8070246 31284512 PMC6678835

[B194] Sheehan D. McDonagh B. (2020). The clinical potential of thiol redox proteomics. Expert Review Proteomics 17 (1), 41–48. 10.1080/14789450.2020.1704260 31826671

[B195] Shin S. Yu J. Tae H. Zhao Y. Jiang D. Qiao Y. (2024). Exploring the membrane-active interactions of antimicrobial long-chain fatty acids using a supported lipid bilayer model for gram-positive bacterial membranes. ACS Applied Materials & Interfaces 16 (42), 56705–56717. 10.1021/acsami.4c11158 39388376

[B196] Shirke M. A. Magdum C. S. Patki S. R. Disouza J. (2014). Quality standardization and toxicity study of garlic on a cardiovascular formulation. Int. J. Bioassays 3 (10), 3363–3369.

[B197] Song X. Yue Z. Nie L. Zhao P. Zhu K. Wang Q. (2021). Biological functions of Diallyl disulfide, a garlic-derived natural organic sulfur compound. Evidence-based Complementary Alternative Medicine eCAM 2021, 5103626. 10.1155/2021/5103626 34745287 PMC8570849

[B198] Song F. Verheust Y. Sampers I. Raes K. (2025). The stability of isothiocyanates in broccoli extract: oxidation from erucin to sulforaphane was discovered. Food Chem. 480, 143872. 10.1016/j.foodchem.2025.143872 40120311

[B199] Stoian I. A. Vlad A. Gilca M. Dragos D. (2025). Modulation of Glutathione-S-Transferase by phytochemicals: to activate or inhibit is the question. Int. Journal Molecular Sciences 26 (15), 7202. 10.3390/ijms26157202 40806333 PMC12346029

[B200] Suleyman M. (2025). Sulforaphane as a feed additive: histopathological and immunomodulatory effects in broiler chickens. Alexandria Sci. Exch. J. 46, 603–611. 10.21608/asejaiqjsae.2025.445013

[B202] Tang K. (2020). Chemical diversity and biochemical transformation of biogenic organic sulfur in the. Ocean 7, 68. 10.3389/fmars.2020.00068

[B203] Tang Y. Li F. Gu D. Wang W. Huang J. Jiao X. (2021). Antimicrobial effect and the mechanism of diallyl trisulfide against *Campylobacter jejuni* . Antibiot. 10 (3), 246. 10.3390/antibiotics10030246 33801353 PMC7999961

[B204] Tang Y. Lv D. Tao Y. Wang J. (2025). The therapeutic effects of natural organosulfur compounds on atherosclerosis and their potential mechanisms: a comprehensive review. Front. Cardiovascular Medicine 12, 1599154. 10.3389/fcvm.2025.1599154 40666422 PMC12259706

[B205] Tedeschi P. Nigro M. Travagli A. Catani M. Cavazzini A. Merighi S. (2022). Therapeutic potential of Allicin and aged garlic extract in alzheimer’s disease. Int. J. Mol. Sci. 23 (13), 6950. 10.3390/ijms23136950 35805955 PMC9266652

[B206] Teixé-Roig J. Oms-Oliu G. Odriozola-Serrano I. Martín-Belloso O. (2023). Emulsion-Based delivery systems to enhance the functionality of bioactive compounds: towards the use of ingredients from natural, sustainable sources. Foods 12 (7), 1502. 10.3390/foods12071502 37048323 PMC10094036

[B207] Thakur A. Mikkelsen H. Jungersen G. (2019). Intracellular pathogens: host immunity and microbial persistence strategies. J. Immunology Research 2019, 1356540. 10.1155/2019/1356540 31111075 PMC6487120

[B208] Thakur P. Dhiman A. Kumar S. Suhag R. (2024). Garlic (Allium sativum L.): a review on bio-functionality, allicin's potency and drying methodologies. South Afr. J. Bot. 171, 129–146. 10.1016/j.sajb.2024.05.039

[B210] Ulasov A. V. Rosenkranz A. A. Georgiev G. P. Sobolev A. S. (2022). Nrf2/Keap1/ARE signaling: towards specific regulation. Life Sciences 291, 120111. 10.1016/j.lfs.2021.120111 34732330 PMC8557391

[B211] Unoki T. Akiyama M. Kumagai Y. (2020). Nrf2 activation and its coordination with the protective defense systems in response to Electrophilic stress. Int. J. Mol. Sci. 21 (2), 545. 10.3390/ijms21020545 31952233 PMC7013553

[B213] van Steenwijk H. P. Vinken A. van Osch F. H. M. Peppelenbos H. Troost F. J. Bast A. (2023). Sulforaphane as a potential modifier of calorie-induced inflammation: a double-blind, placebo-controlled, crossover trial. Front. Nutrition 10, 1245355. 10.3389/fnut.2023.1245355 38089924 PMC10713815

[B214] Vaou N. Stavropoulou E. Voidarou C. Tsigalou C. Bezirtzoglou E. (2021). Towards advances in medicinal plant antimicrobial activity: a review Study on challenges and future perspectives. Microorganisms 9 (10), 2041. 10.3390/microorganisms9102041 34683362 PMC8541629

[B216] Vigneron A. Cruaud P. Culley A. Couture R. Lovejoy C. Vincent W. (2021). Genomic evidence for sulfur intermediates as new biogeochemical hubs in a model aquatic microbial ecosystem. Microbiome 9, 46. 10.1186/s40168-021-00999-x 33593438 PMC7887784

[B217] Wang X. Fu H. Y. He W. Xiang Y. T. Yang Z. C. Kuang Y. (2022). Synthesis and antibacterial activity evaluation of biphenyl and Dibenzofuran derivatives as potential antimicrobial agents against antibiotic-resistant bacteria. Curr. Issues Molecul. Bio. 44 (9), 4087–4099. 10.3390/cimb44090280 36135192 PMC9497828

[B218] Wardecki D. Dołowy M. Bober-Majnusz K. (2023). Assessment of lipophilicity parameters of antimicrobial and immunosuppressive compounds. Mol. 28 (6), 2820. 10.3390/molecules28062820 36985792 PMC10059999

[B219] Waskito L. A. Rezkitha Y. A. A. Vilaichone R. K. Wibawa I. D. N. Mustika S. Sugihartono T. (2022). Antimicrobial resistance profile by metagenomic and metatranscriptomic approach in clinical practice: opportunity and challenge. Antibiot. 11 (5), 654. 10.3390/antibiotics11050654 35625299 PMC9137939

[B220] Wlosinska M. Nilsson A. C. Hlebowicz J. Hauggaard A. Kjellin M. Fakhro M. (2020). The effect of aged garlic extracts on the atherosclerotic process - a randomized double-blind placebo-controlled trial. BMC Complement. Med. Ther. 20 (1), 132. 10.1186/s12906-020-02932-5 32349742 PMC7191741

[B221] Wlosinska M. Nilsson A. C. Hlebowicz J. Fakhro M. Malmsjö M. Lindstedt S. (2021). Aged garlic extract reduces IL-6: a double-blind placebo-controlled trial in females with a low risk of cardiovascular disease. Evid Based Complement Alternat Med. 2021, 6636875. 10.1155/2021/6636875 33868439 PMC8032523

[B222] Wu G. Baumeister R. Heimbucher T. (2023). Molecular mechanisms of lipid-based metabolic adaptation strategies in response to cold. Cells 12 (10), 1353. 10.3390/cells12101353 37408188 PMC10216534

[B223] Xie Y. Chen J. Wang B. Peng A.-Y. Mao Z.-W. Xia W. (2022). Inhibition of quorum-sensing regulator from *Pseudomonas aeruginosa* using a flavone derivative. Molecules 27 (8), 2439. 10.3390/molecules27082439 35458637 PMC9031925

[B224] Yamamoto M. Kensler T. W. Motohashi H. (2018). The KEAP1-NRF2 System: a thiol-based sensor-effector apparatus for maintaining redox homeostasis. Physiol. Reviews 98 (3), 1169–1203. 10.1152/physrev.00023.2017 29717933 PMC9762786

[B225] Yan L. Yan Y. (2023). Therapeutic potential of sulforaphane in liver diseases: a review. Front. Pharmacol. 14, 1256029. 10.3389/fphar.2023.1256029 37705537 PMC10495681

[B226] Yang W. Liu P. Chen Y. Lv Q. Wang Z. Huang W. (2022). Dictamnine inhibits the adhesion to and invasion of uropathogenic *Escherichia Coli* (UPEC) to urothelial cells. Molecules 27 (1), 272. 10.3390/molecules27010272 35011504 PMC8746591

[B227] Yeoman C. J. Fields C. J. Lepercq P. Ruiz P. Forano E. White B. A. (2021). *In vivo* competitions between *Fibrobacter succinogenes, Ruminococcus flavefaciens,* and *Ruminoccus albus* in a gnotobiotic sheep model revealed by multi-omic analyses. mBio 12 (2), e03533. 10.1128/mBio.03533-20 33658330 PMC8092306

[B228] Yuan J. M. Kensler T. W. Dacic S. Hartman D. J. Wang R. Balogh P. A. (2025). Randomized phase II clinical trial of sulforaphane in former smokers at high risk for lung cancer. Cancer Prevention Research 18 (6), 335–345. 10.1158/1940-6207.CAPR-24-0386 40041932 PMC13321263

[B229] Zakharova L. Y. Maganova F. I. Sinyashin K. O. Gaynanova G. A. Mirgorodskaya A. B. Vasilieva E. A. (2023). Supramolecular strategy for the design of nanocarriers for drugs and natural bioactives: current State of the art (A review). Russ. J. General Chem. 93, 1867–1899. 10.1134/s1070363223070253

[B230] Zambrano V. Bustos R. Mahn A. (2019). Insights about stabilization of sulforaphane through microencapsulation. Heliyon 5 (11), e02951. 10.1016/j.heliyon.2019.e02951 31844781 PMC6895643

[B231] Zhang C. L. Zeng T. Zhao X. L. Xie K. Q. (2013). Garlic oil attenuated nitrosodiethylamine-induced hepatocarcinogenesis by modulating the metabolic activation and detoxification enzymes. Int. J. Bio. Sci. 9 (3), 237–245. 10.7150/ijbs.5549 23494807 PMC3596709

[B232] Zhang H. Shang C. Tian Z. Amin H. K. Kassab R. B. Abdel Moneim A. E. (2020). Diallyl disulfide suppresses inflammatory and oxidative machineries following Carrageenan injection-induced paw edema in mice. Mediat. Inflammation 2020, 8508906. 10.1155/2020/8508906 32377166 PMC7180418

[B233] Zhang S. Wang J. Ahn J. (2023). Advances in the discovery of efflux pump inhibitors as novel potentiators to control antimicrobial-resistant pathogens. Antibiot. 12 (9), 1417. 10.3390/antibiotics12091417 37760714 PMC10525980

[B234] Zhang Y. B. Wang J. F. Wang M. X. Peng J. Kong X. D. Tian J. (2024). Nano-based drug delivery systems for active ingredients from traditional Chinese medicine: harnessing the power of nanotechnology. Front. Pharmacol. 15, 1405252. 10.3389/fphar.2024.1405252 38910887 PMC11190311

[B235] Zhang L. Li T. Liu J. Sun J. Niu J. Ren D. (2025). The regulation of the NF-κB p65 and Nrf2/HO-1 signaling pathways by fucoxanthin in Human THP-1 Monocyte macrophages under a lipopolysaccharide-induced inflammation model. Foods 14 (10), 1746. 10.3390/foods14101746 40428524 PMC12110976

[B236] Zhang S. Chen W. Zhou J. Liang Q. Zhang Y. Su M. (2025). The benefits and safety of monoclonal antibodies: implications for cancer immunotherapy. J. Inflamm. Res. 18, 4335–4357. 10.2147/JIR.S499403 40162076 PMC11952073

[B237] Zhao X. Cheng T. Xia H. Yang Y. Wang S. (2024). Effects of garlic on glucose parameters and lipid profile: a systematic review and meta-analysis on randomized controlled trials. Nutrients 16 (11), 1692. 10.3390/nu16111692 38892625 PMC11174586

[B238] Zhou S. Yan X. Qiao X. Qiu Z. Zhu W. Lu X. (2025). Evaluate the stability of synthesized allicin and its reactivity with endogenous compounds in garlic. NPJ Science Food 9 (1), 18. 10.1038/s41538-025-00374-2 39915544 PMC11802734

[B239] Zhuang H. H. Chen Y. Hu Q. Long W. M. Wu X. L. Wang Q. (2023). Efficacy and mortality of ceftazidime/avibactam-based regimens in carbapenem-resistant Gram-negative bacteria infections: a retrospective multicenter observational study. J. Infection Public Health 16 (6), 938–947. 10.1016/j.jiph.2023.04.014 37087853

